# Methodological Design Choices Can Affect Air Pollution Exposure Disparity Estimates: A Case Study on California’s Agricultural Sector

**DOI:** 10.1021/acs.est.5c10796

**Published:** 2026-02-05

**Authors:** Libby H. Koolik, Simone Speizer, Clara Rong, Sarah Chambliss, Julian D. Marshall, Rachel Morello-Frosch, Christopher W. Tessum, Joshua S. Apte

**Affiliations:** † Department of Civil and Environmental Engineering, 1438 University of California, Berkeley, Berkeley, California 94720, United States; ‡ Energy and Resources Group, 1438 University of California, Berkeley, Berkeley, California 94720, United States; § Department of Civil and Environmental Engineering, Massachusetts Institute of Technology, Cambridge, Massachusetts 02139, United States; ∥ Department of Population Health, 12330 University of Texas at Austin Dell Medical School, Austin, Texas 78712, United States; ⊥ Department of Civil and Environmental Engineering, University of Washington, Seattle, Washington 98195, United States; # Department of Environmental Science, Policy, and Management, 1438 University of California, Berkeley, Berkeley, California 94720, United States; ∇ School of Public Health, 1438 University of California, Berkeley, Berkeley, California 94720, United States; ○ Department of Civil and Environmental Engineering, 14589 University of Illinois at Urbana−Champaign, Urbana, Illinois 61801, United States

**Keywords:** environmental justice, PM_2.5_, distributional justice, agriculture, equity

## Abstract

People of color in the United States are disproportionately and unfairly exposed to air pollution. Equity-oriented scientific evaluations quantifying these disparities often use population-average exposure metrics to capture the overall inequality within a system. Utilizing these metrics involves choices about the exposure input for assessing disparity, the study geography, and the reference population, which are critical to understanding disparities and effectively designing interventions. Here, we use a case study of exposure to fine particulate matter (PM_2.5_) from California’s agricultural sector to dissect the implications of these decisions. Using a reduced-complexity model and emissions of PM_2.5_ and precursors, we compare estimates of racial and ethnic disparities in exposure resulting from different combinations of these methodological choices. The full population distributions highlight differences between disparities at the extremes (e.g., 90th percentile) and at the mean. Additionally, the selection of study geography and reference population can influence the magnitude and relative ordering of exposure disparities. Thus, methodological choices can lead to different conclusions for the same concentration and population surfaces; this can impact not only the findings of an individual study but also have implications for mitigation strategies. We conclude with recommendations for best practices for making, justifying, and communicating these methodological decisions.

## Graphical abstract

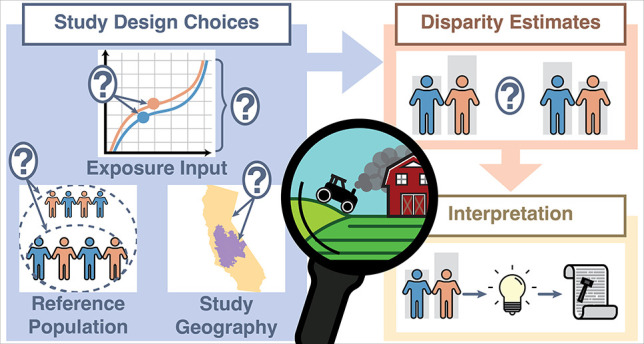

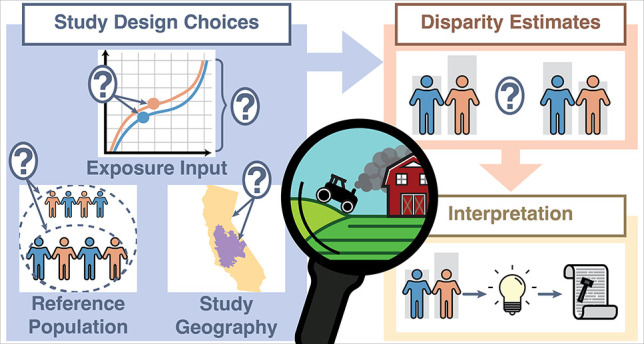

## 1 Introduction

Across the United States and within California, people of color are disproportionately exposed to fine particulate matter (PM_2.5_).^1,2^ These disparities stem from decades of racially discriminatory policies.^3,4^ Fully addressing the ongoing disparities requires consideration of key tenets of environmental justice, including (but not limited to) distributive, procedural, and recognitional justice.^5–8^ Quantitative research is often best positioned to evaluate distributive equity concerns, though procedural and recognitional issues often arise in the framing and development phases of such research questions, and should not be neglected.^6^

However, research is only useful to the extent that a given study’s design is consistent with the question it aims to answer. As the research community continues to advance our understanding of air pollution exposure disparities, it is worthwhile to step back and explore the interplay between the questions we ask and the methods we use to answer them. Past reviews of the environmental justice literature have highlighted the variance in conclusions resulting from diverging methodological choices, particularly in studies employing unit-hazard coincidence or proximity-based techniques.^9^ It is valuable to extend a consideration of these issues to new modeling techniques used in exposure disparity assessments, especially as the scientific community has recently called for improved quantification and tracking of exposure disparities.^10–13^ Here, we explore how key methodological choices can shape our understanding of distributive exposure disparities and for whom interventions are most needed. We aim to provide quantitative evidence to build upon equity-oriented research frameworks and recommendations across exposure science.^5–7,9,14,15^ Ultimately, the choices of where and how to intervene may—intentionally or unintentionally—be prompted by a particular framing of an equity question and associated study design considerations. Using a case study of exposure to agricultural emissions in California, we demonstrate the range of potential exposure disparity conclusions arising from design choices.

Disparities in exposure and health are well-documented at the national scale and within states like California.^1,2,4,16–20^ Much of the quantitative literature characterizing disparities and their solutions focuses on a single summary statistic (e.g., mean, median) relative to a large reference population (e.g., state-level, national). Less common is a holistic examination of the *distribution* of outcomes across the full geographic boundary or across spatial scales. Within the literature, there are two main categories of disparity indicators (Figure 1).^7^ The first summarizes the overall inequality within a population (e.g., Atkinson Index, Gini Index, and Dissimilarity Index).^20–25^ These metrics provide a system-wide statistic to capture overall inequality (e.g., deviations from exposures being equal for all people) but do not identify which groups are more or less exposed. A second category of indicators are “per demographic group”; one can compare these statistics across groups to assess between-group disparities.^2,21,26–28^ For these metrics, exposures across a specific group are commonly summarized using the population-weighted mean (PWM, the average exposure for members of a specific group) or point estimates of other population-weighted statistics (e.g., 75th percentile exposure). From the PWM or distribution point estimates, disparity can be estimated as an “exposure gap” (e.g., difference between most and least exposed groups; sometimes referred to as an “inequality”) or as an absolute (difference between group and total population; units: μg/m^3^) or relative (percent difference between a group and total population; units: % difference) disparity.^2,4,11,21,22,26,28–31^ Disparity at the PWM is often used to identify which interventions will yield the highest reduction in exposure or disparity overall, thereby neglecting heterogeneity within groups in exchange for a focus on achieving equality across the means.^10,11,26^ As shown in Koolik et al.,^10^ the absolute disparity can be decomposed into changes in emissions, average exposure, and relative disparity, which can illuminate potential policy avenues for mitigating disparities. These discrete estimates can facilitate intergroup comparisons but may fail to capture important intragroup variation. Thus, another approach for evaluating equity is the determination of demographic differences along the exposure distribution.^32–34^ While these metrics provide different options for evaluating disparity and inequality, they do not themselves address the question of what a fair distribution of exposures would be. Establishing a framework for justice and developing solutions aligned with this framework that dismantle the underlying systemic biases and unfair treatment requires socially grounded and intersectional analyses beyond what we consider here.^35^

**Figure 1:**
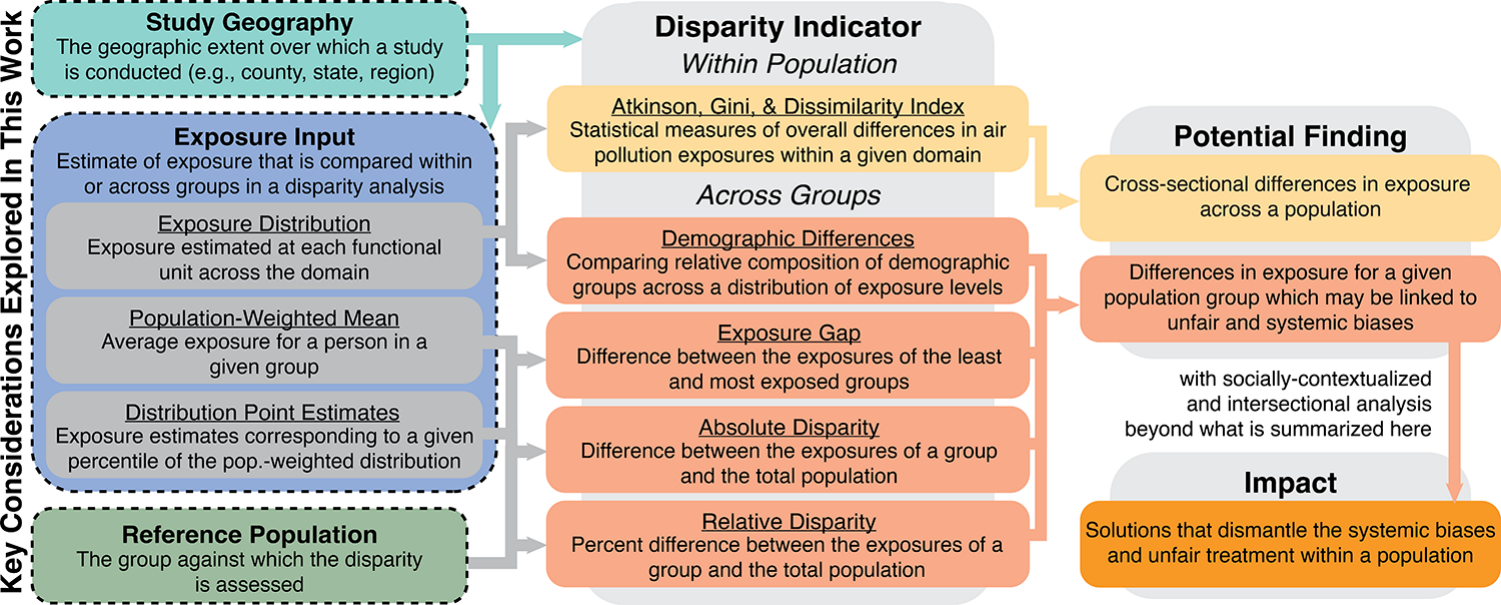
Definitions and relationships between terms for the key study considerations explored here, disparity indicators, potential findings, and impacts. The three methodological design considerations that are the main focus of this paper are bolded and circled in dashed lines. We define three different exposure input options: the exposure distribution, population-weighted mean, and distribution point estimates. We illustrate how these design choices relate to various disparity indicators and the potential findings. All relevant terms used in this work are underlined and defined.

In the past decade, the literature has increasingly recognized and quantified the equity-oriented outcomes of environmental policies.^28,30,33,34,36–38^ The advent of high-resolution modeling tools has further facilitated the estimation of the exposure disparities. In the air pollution modeling literature, the PWM, absolute disparity, and relative disparity have emerged as some of the most common metrics for quantifying progress toward advancing environmental equity. Some studies have reviewed different inequality metrics and modeling tools.^7,22,24,39–94^ Relatively less attention has been paid to the role of the choices of geographic extent and reference population that underlie these metrics, particularly in the context of air pollution exposure disparity.^9,42,43^

There are several reasons why the PWM and disparity derived from it might not be ideal for a given question. For example, the PWM neglects exposure variability across space that covaries with underlying health vulnerabilities and population dynamics.^44–48^ Accordingly, disparities at the mean often differ from disparities at the extremes. Thus, failure to characterize how exposures at the high extremes compare across groups could incompletely assess inequity.

Additionally, per-group disparity estimates rely on a reference population against which to compare against. When seeking to compare disparities among groups in overexposed areas, a comparison among only groups in those overexposed areas may miss important regional or statewide contexts. The choices of the reference population and geographic domain over which to evaluate these disparities are thus fundamental considerations. In our exploration of these variables, we build upon the insights from Mohai et al.^9^ regarding the diverging conclusions that can arise when using different geographic units of analysis. Figure 1 highlights these three key study design choices (exposure input, reference population, and study geography), their role in calculating disparity indicators, and the conclusions that can be drawn from these analyses. Two additional related considerations that have already been explored elsewhere in the literature are the choice of modeling tool and its spatial resolution.^14,29^

To illustrate the impact of these methodological considerations on addressing different equity-oriented questions, we use a case study of inequity in exposure to PM_2.5_ concentrations resulting from California’s agricultural sources. We focus on agricultural sources in California as an example case in which the variability in conclusions for a single sector’s impacts on equity should be striking. Although nationally, agriculture is not considered a major driver of exposure inequality for people of color, prior studies in California suggest that the agricultural sector could have disparate impacts as large as other major emitters, such as industrial sources and on-road vehicles.^2,27^ Furthermore, agricultural emissions are estimated to cause over 2000 excess deaths per year in California.^49^ One difference between agriculture and other emitting sectors is the spatial distribution of these sources, as agricultural land and emitting facilities are concentrated in a relatively small portion of the state, while also representing a more diffuse and rural source than many others (Figure S1). Moreover, because many agricultural production areas overlap substantially with regions of air pollution nonattainment (e.g., California’s San Joaquin Valley), mitigation of this sector may play a major role in state implementation planning.^50^ Beyond emissions, there are other dimensions that drive overall health inequities in this case study. Agricultural facilities and farm lands have been a historical driver of both immigration to California from Hispanic America and many socioeconomic and environmental challenges, causing a number of intersectional equity issues and stressors.^51–56^ High underlying health risk factors in farmside communities could further amplify the impact of exposure disparities on overall health inequities. Thus, agriculture provides a policy-relevant case study for a careful and thorough investigation of how study design considerations can influence the quantification of exposure disparities.

## 2 Methods

We model PM_2.5_ exposure and disparity associated with emissions from California’s agricultural sector. Spatially allocated primary PM_2.5_, oxides of nitrogen (NO_*x*_), ammonia (NH_3_), oxides of sulfur (SO_*x*_), and volatile organic compounds (VOC) emissions from California’s agricultural sector are estimated from the 2014 National Emissions Inventory.^57^ We use the Intervention Model for Air Pollution (InMAP), a reduced-complexity model, to estimate annual average concentrations of total PM_2.5_ from agricultural sources across California along a population-weighted irregular grid.^34,58^ American Community Survey 5-year population estimates for 2012−2016 are used to estimate Census block group (BG) demographic exposures.^59^ For our disparity assessment, we consider Hispanic people of any race as “Hispanic”; all other racial-ethnic groups include only those who are non-Hispanic (e.g., “Black” refers to Non-Hispanic Black Californians), consistent with other literature in this field.^3,21,34^

We compare disparity estimates that result from three axes of methodological choices. First, we estimate the full distribution of BG level PM_2.5_ concentrations (*n* = 23,192 BGs) using the population of each major racial-ethnic category statewide. Second, we compare our findings across four study geographies in California: (1) statewide, (2) Agricultural Areas, (3) the San Joaquin Valley, and (4) Overall High PM_2.5_ Exposure Areas in California (Figure S2). The three substatewide study geographies are defined by finding BGs that meet a specific criterion. For Agricultural Areas, we select all BGs that have at least 25% of their land area overlapping with at least two of the three independently derived agriculturally relevant data sets from the California government.^60–62^ The San Joaquin Valley contains all block groups that overlap with the San Joaquin Valley Air District. Unlike all other modeling in this study, the Overall High PM_2.5_ Exposure Areas domain considers PM_2.5_ from all potential sources, as it is derived by finding all BGs in the top 25th percentile using two observationally constrained data sets of total PM_2.5_ concentration for 2014.^63,64^ Additional details and intermediate spatial extents are included in Figure S2. Finally, we use the four study geographies to test the choice of reference population by comparing disparity metrics relative to a comparison group either within a region or statewide.

Each of these four geographies has distinct policy relevance. The statewide domain represents a typical and governable spatial extent for this type of equity-oriented study.^28,34^ The Agricultural Areas, which are selected by combining three data sets about land use in California, represent an analytical focus on high emission areas from this sector.^60–62^ The San Joaquin Valley air district is surrounded by an air basin that has been designated in nonattainment for decades due, in part, to substantial agricultural activity. Finally, the Overall High PM_2.5_ exposure area case provides a counterexample of a region that is not selected for any direct tie to agriculture. All geospatial analysis is conducted in Python using open-source geospatial and data science packages (e.g., geopandas, pandas, numpy; see the SI for specific versions and details).

## 3 Results and Discussion

### 3.1 Disparity along the Distribution Can Differ from Disparity at the Mean

In Figure 2, we compare the distribution of modeled total PM_2.5_ exposure resulting from agricultural sector emissions for the Hispanic, Black, and total population (all racial-ethnic groups in Figure S3). Many areas of high population density and concentration are along California’s San Joaquin Valley (Figure S4), consistent with the spatial pattern of agricultural emissions (Figure S1). Based on the National Emissions Inventory for all sources across California, agricultural activities are responsible for ∼80% of all statewide NH_3_ emissions and <5% of statewide primary PM_2.5_, NO_*x*_, SO_*x*_, and VOC emissions (Figure S1).^56^ In Figure 2a, the PWMs (circles) from agriculture alone are superimposed on top of box plots, which demonstrate the ranges in exposure levels. For example, while the total PWM exposure to PM_2.5_ from agricultural emissions for the full population is 1.5 μg/m^3^, the top 25th percentile of Californians are exposed to concentrations of 2.1 μg/m^3^ or higher. As shown in Figure 2a, based on the PWM, Hispanic and Black Californians are disproportionately exposed, with disparities relative to the statewide PWM (stars) of 14% and 5%, respectively. All other groups considered here are not overburdened relative to the statewide average population (Figure S3). Hispanic people in California experience the highest concentrations of agricultural PM_2.5_ at the PWM (1.7 μg/m^3^), likely arising from the historical legacy of farmworker demographics in the state (i.e., the large share of California farmworkers that are of Hispanic origin).^56^

**Figure 2:**
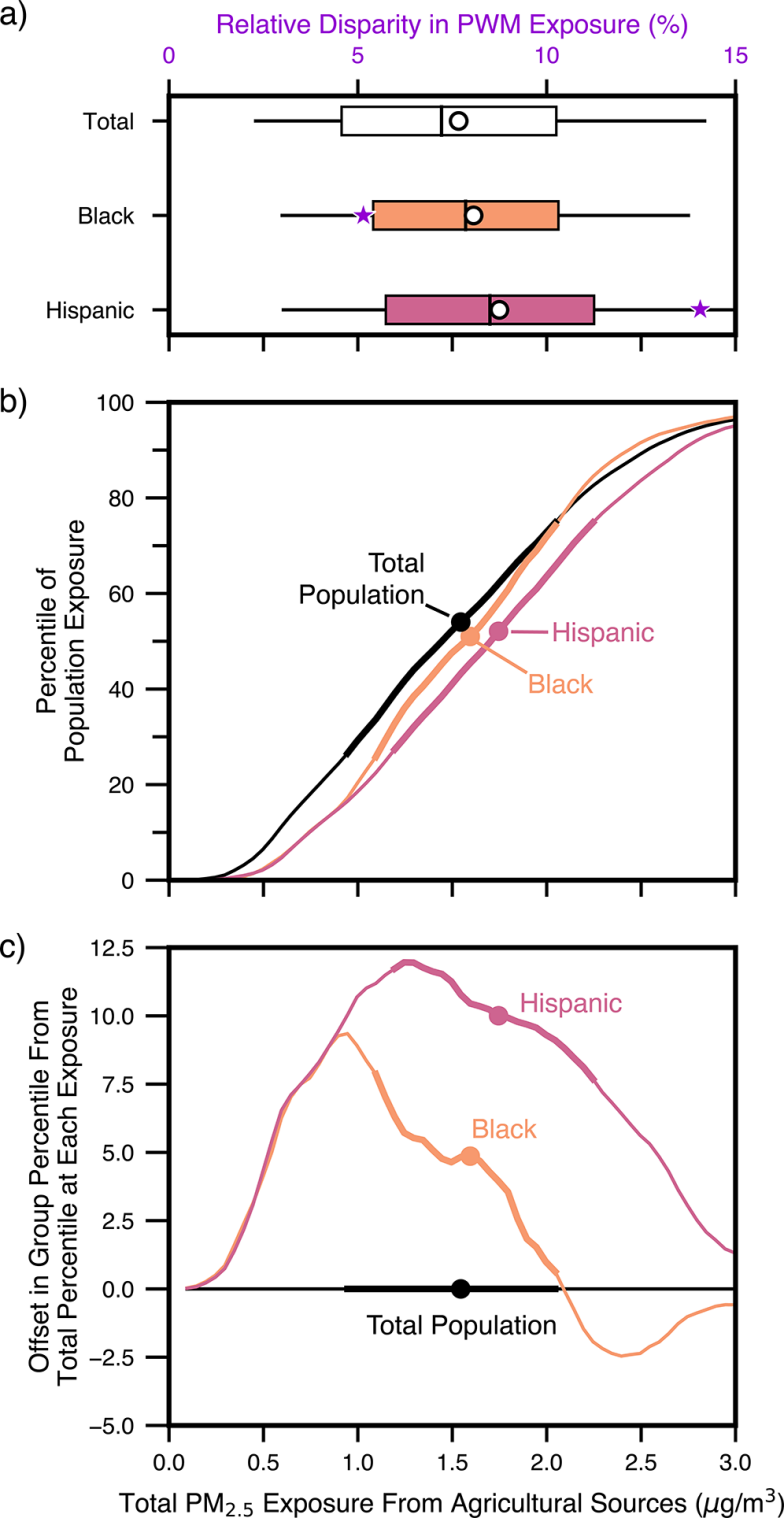
Distributions of PM_2.5_ concentrations across California by race-ethnicity provide a more nuanced understanding of disparities than a simple mean. Here, we highlight results for the Black, Hispanic, and total populations; see Figure S3 for results for all racial-ethnic groups. (a) Box plots demonstrate the range of block group exposures by race-ethnicity compared to the total population. The symbols on the box plot per group are as follows: the PWM is a circle, the median is a bar, the interquartile range is the box, and the fifth/95th percentile are the whiskers. Superimposed on each box is a purple star representing the relative disparity in exposure estimated at the PWM (corresponding to the top *x*-axis). (b) Here, we show distribution curves for binned PM_2.5_ exposures (bin size = 0.05 μg/m^**3**^). The thicker lines represent exposures in the interquartile range. We truncate the figure at 3 μg/m^**3**^, just above the 96th percentile exposure for the total population. Circle markers indicate the PWMs. (c) Each group’s exposure distribution curve is transformed into an offset to demonstrate how absolute exposure disparities evolve across the distribution. The offset represents how much earlier in the percentile distribution a given PM_2.5_ exposure occurs for that group relative to the total population (i.e., for a given PM_2.5_ concentration, the offset is the difference between the total population percentile at that concentration and the group’s percentile at that concentration).

Both Figures 2a and 2b compare disparities in exposure to PM_2.5_ from agricultural emissions across the distribution with those at the PWM by race-ethnicity. If the research question seeks to find the most disparately exposed group overall from agricultural emissions, the identification of Hispanic Californians as this group is accurate at both the PWM and each point noted by the box plots and across the entire distribution. In fact, the full distribution of exposures to PM_2.5_ from agricultural sources for Hispanic Californians is translated toward higher concentrations relative to the statewide distribution and the distributions of every other group (Figure 2b). In Figure 2c, we visualize this translation by plotting the offset in the group percentile from the curve in Figure 2b at each PM_2.5_ concentration. A simple interpretation of this figure is that points above zero represent that a given exposure level occurs earlier in the distribution; points below zero represent the opposite. For example, the largest offset (∼12 percentile points) for Hispanic Californians occurs at approximately 1.3 μg/m^3^. When comparing what percent of each population group is exposed to at least 1.3 μg/m^3^ of agriculture-related PM_2.5_ concentrations, the offset demonstrates that 12% *more* of the Hispanic population exceeds this threshold.

A different story unfolds for the Black population. While Black Californians have the second highest PWM exposure to PM_2.5_ from agricultural sources (Table S1), the 75th percentile exposure is nearly identical to that of the total population distribution (2.1 μg/m^3^), and the 95th percentile exposure is slightly lower than that of the total population (Black population 3% lower than total population exposure of 2.8 μg/m^3^). Just after the 75th percentile, Black Californians are no longer disproportionately exposed, relative to the same percentile of the total statewide population (represented by crossing zero in Figure 2c, and the crossing of lines in Figure 2b). This nuance at the tails of the distributions is lost when describing exposures with only a mean-based summary statistic but is incredibly important given the spatial variability in other health-relevant risk factors (e.g., other underlying health conditions, vulnerability, and age structure). Thus, if the research question aims to characterize whether or not Black Californians are disproportionately exposed to PM_2.5_ from agricultural sources, the PWM does not provide a complete answer.

The differences in trends for Hispanic and Black Californians result from the confluence of population and exposure distributions in space (Figure S5). BGs with larger populations of Hispanic residents tend to have a higher concentration of total PM_2.5_ from agricultural sources (Figure S5b). Yet, little-to-no trend exists for Black Californians. In an alternative framing, we can also understand this by noting that the highest exposure BGs are disproportionately high in their Hispanic population (Figure S5c); within the top 25th percentile of exposures, 53% of people are Hispanic, as compared to 39% of the statewide population. In contrast, the Black population share among these highest exposed BGs is approximately the same as its share statewide (6%).

### 3.2 Choices of Reference Population and Study Geography Can Substantially Affect Conclusions

The variation across space in both the exposures to PM_2.5_ from agricultural sources and populations suggests that the choice of spatial domain has the potential to substantially affect what the equity-oriented question is being answered. We compare disparities for Hispanic and Black Californians across all four study geographies in Figure 3 (version with all groups in Figure S6). The shares of the population and cumulative PM_2.5_ population exposure (defined as the product of BG population and exposure) in each of these domains are shown in Figure 3a.^65^

**Figure 3:**
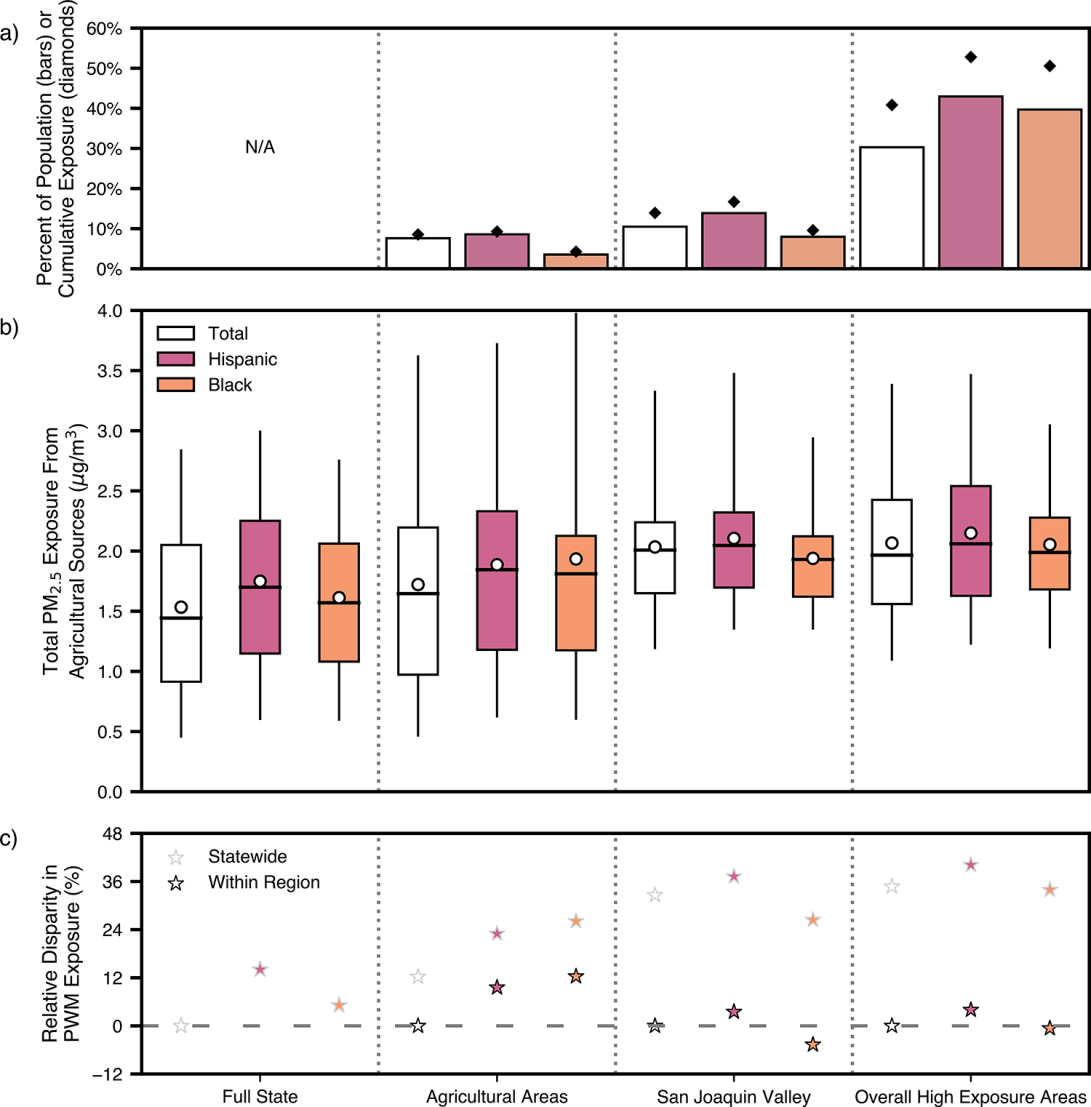
Exposure and disparity for Hispanic and Black Californians using four different geographic domains. We showcase the different results that can arise from the same data, analyzed across four study geographies, each with two different reference populations. Results for the total, Hispanic, and Black populations are shown from left to right, respectively. (a) The percent of the overall state population is shown as a bar, with the percent of cumulative population exposure from agricultural emissions (sum of concentrations multiplied by population at each block group) as a diamond. For the Full State domain, the percent of population and exposure is 100%, so this is not shown. (b) Box plots of exposure distributions are drawn for each group in each region, consistent with Figure 2a. (c) The relative disparity is calculated for each group relative to the total population statewide (lighter stars) and within that region (darker stars). In all cases, the relative disparity compared to statewide disparity is higher than the relative disparity when calculated within the geographic region. Hispanic Californians are disparately exposed in all of their study geographies. Whether Black Californians are disparately exposed within the region of interest changes depending on the geographic domain.

Figure 3b highlights how methodological choices can drive a range of conclusions from the same data set. Within each region, Hispanic Californians are often—but not always—the group most exposed to PM_2.5_ from agricultural emissions at the PWM. For example, in the Overall High Exposure Areas, the PWM exposure for the Hispanic population is 4% higher than the PWM for the total population in that region. The exception is in Agricultural Areas, where the PWM is highest for Black Californians. In fact, a key finding of this analysis is that this choice of geographic domain can result in starkly divergent conclusions for the Black population. Depending on the domain, Black Californians face either lower exposure (San Joaquin Valley), nearly equal exposure (Overall High Exposure Areas), or the highest exposure (Agricultural Areas), when evaluating disparities at the PWM internal to each of these regions, as shown in Figure 3c.

From an analysis considering only the Agricultural Areas, one might simply conclude that agriculture is an important contributor to the disproportionate exposure for Black Californians statewide. Rather, the Black population *located in these areas* are disproportionately exposed to agricultural PM_2.5_ as compared to other groups *in this area*. This nuance is tremendously important, as a statewide policymaker aiming to reduce the disparate exposures faced by California’s overall Black population may see relatively little impact from mitigating agricultural sources.

Furthermore, investigating why the Black population has the highest exposure in Agricultural Areas uncovers important underlying patterns and reinforces how the choice of geographic domain can matter. First, only 4% of the total statewide Black population resides in Agricultural Areas (Figure 3a). Second, of the Black population in those areas, an outsized portion is present in a single BG in Chino, CA. (There are 1,504 BGs in Agricultural Areas, so on average each BG contains 0.07% (i.e., 1/1,504) of the Black population. This BG contains 3.5% of the Black population in Agricultural areas.) Exposure to agricultural sources in that BG is relatively high (6.9 [this BG] vs 1.6 [spatial average in all Agricultural Areas] μg/m^3^, Figure S7). This BG includes the California Institution for Men, whose incarcerated people account for 30% of the BG’s total population. While specific demographic information for the California Institution for Men is not available, Black men constitute approximately 30% of incarcerated men statewide.^66^ The intersection of high air pollution exposures and racial disparities in incarceration rates, while not the major focus of this work, is an important area of research and activism (e.g., the Prison Ecology Project and the Toxic Prisons Mapping Project).^67–72^

Evaluating the dynamics occurring in one BG is useful for understanding population distributions, but use of reduced-complexity modeling tools to do so may introduce additional uncertainties. As the sample size of BGs decreases with smaller geographic domains, the imprecision of reduced-complexity air pollution modeling tools can increasingly impact the conclusions. For investigating concentrations in a specific BG, alternatives to RCMs include empirical models and satellite-based estimates.^21,29,73–76^ Regardless of the modeling tool or data source, investigating the full distribution of populations and exposures can shed light on potential outliers that impact overall conclusions. In our case study, for example, comparing median exposures (bars in Figure 3b) for the Black and Hispanic populations in Agricultural Areas tells a different story than comparing the PWMs. Median exposures are not affected by extreme values. In Agricultural Areas, for example, the median exposure is higher for Hispanic individuals than for Black individuals.

The disparities experienced by the Hispanic and Black populations within these geographies relative to the total statewide population are dramatic (Figure 3c). The difference in magnitude of disparities arises because PWM exposures from agricultural sources are higher than those statewide in each of the spatial domains. The increase is particularly stark in the San Joaquin Valley and the Overall High PM_2.5_ Exposure Areas (total population PWMs exceeding 2 μg/m^3^ from agricultural sources alone). Put simply as an example, the exposure for Hispanic residents of Overall High PM_2.5_ Areas, as shown in Figure 3c and Table S1, is slightly higher than that of all people residing in these areas (4%), but significantly higher than all Californians on average (40%). By limiting the analysis is limited to differences across groups within highly polluted regions, the magnitude of the disparities is much smaller. When we instead consider the full statewide context, we see that the disparities relative to the statewide average are much larger. Choosing a reference population of the least exposed group statewide further exacerbates these reported disparities (Table S1). In the San Joaquin Valley and the Overall High Exposure Areas, the PWM exposure for the Hispanic population is only 4% higher than that of the total population within each respective region, and the Black population’s PWM is lower than that of the region’s total population (5% and 1% lower in the San Joaquin Valley and the Overall High Exposure Areas, respectively). The interquartile ranges in these regions are also narrower than those in the full state. In contrast, the tails of the distributions on the upper end are slightly elongated, reflecting the elevated relative presence of high agricultural exposures in these regions. In the Agricultural Areas, within-region disparities are slightly higher, at 10% and 12% for the Hispanic and Black populations, respectively. For the Hispanic population, this is a lower relative disparity at the PWM than that of the statewide analysis (14%); for the Black population, it is higher (up from 5%).

Focusing in further on the Agricultural Areas, we find the highest extreme (95th percentile) exposures in this region, exceeding 3.5 μg/m^3^ for all three groups shown in Figure 3. However, this region also displays the widest range between the extreme low (fifth percentile) and extreme high (95th percentile) exposures for all groups (e.g., for the total population: 3.1 μg/m^3^ in the Agricultural Areas, 2.4 μg/m^3^ in the full state, 2.1 μg/m^3^ in the San Joaquin Valley, and 2.3 μg/m^3^ in the Overall High Exposure Areas).

There are two reasons why limiting the geographic extent to Agricultural Areas does not result in the highest exposures to agriculture across the distribution. First, we define BGs as agricultural based on whether 25% or more of their land area is considered agricultural when compared against three agricultural data sets. However, this does not necessarily mean that the agricultural activities on that land are high emitting. For example, different crops and farming techniques require different levels of fertilizer application and onsite tilling activity. Second, the Agricultural Areas are chosen based on where the *activity* is occurring, not where the *exposures from those activities* are the highest. Given the importance of NH_3_ emissions from agriculture (and therefore the importance of secondary ammonium aerosol production for total PM_2.5_), some of the hotspots in the concentration field occur downwind of the agricultural lands and outside of the Agricultural Areas domain.

The choice to evaluate disparity relative to a comparison group internal to a region or a comparison group on a broader (i.e., statewide) scale depends on the question we want to address. Here, we find that agricultural emissions are responsible for elevating the burden of overall exposure faced by all people living in disproportionately exposed areas (i.e., the San Joaquin Valley and the Overall High PM_2.5_ Exposure Areas), but not for creating substantial intergroup disparities *within* those regions. In contrast, in Agricultural Areas, agricultural emissions both increase overall population exposures and contribute meaningfully to within-region disparities across groups. This nuance matters, as the former suggests spatially targeted but demographically agnostic interventions (e.g., emissions reduction plans), while the latter suggests spatially and demographically targeted interventions (e.g., translated educational materials, targeted filter distribution).

### 3.3 Limitations and Uncertainty

There are several important limitations to our approach. Because we are focusing on a case study of a single sector of exposure in a single state, our specific quantitative insights and the range of possible conclusions could vary across other source categories and/or domains. Still, our qualitative insight that different methodological choices answer different questions is important and should warrant more thorough investigations of population exposures in future studies. Additionally, we repeat our analysis from Figure 3 using observationally constrained, empirically modeled data in Figure S8, and the conclusions from Figure S8 broadly mirror those in Figure 3.

Furthermore, as with many such analyses, we estimate disparities in exposure to outdoor, annual-average ambient concentrations as a surrogate for the true pollution intake.^31,35^ Further work should be done to study the fully time-resolved exposure of these important farmside communities. Additionally, we have limited our equity analysis to exposure disparities, as health outcomes arise from the product of interwoven disparities in exposure and vulnerability due to coexposure to other environmental and social stressors which also vary across space and by race and ethnicity.^77–79^

This work also treats each major racial-ethnic group evaluated as a monolith; in reality, there can be stark differences between individual subpopulations within each of these groups. Incorporating additional demographic characteristics (e.g., immigration status, degree of linguistic isolation, educational attainment) might identify subpopulation groups that are even more systematically overburdened.^80,81^

We employ spatially allocated emissions estimated from the National Emissions Inventory and a reduced-complexity model for our analysis, which enables the high spatial-resolution modeling necessary to understand disparities across a state as large as California at the expense of some fidelity. The specific version of InMAP used for this analysis has been validated and applied in many similar sector-specific analyses.^2,27,30,34,37,49,54^ While there is always the potential for error or bias to propagate through the emissions and model, our qualitative insights are built upon a large sample of gridded concentration data (*n* = 9399) averaged to the Census BG-level (*n* = 23,192 BG) and reported along a 100-point distribution, increasing their robustness to potential outliers and individual grid-cell model bias.

Similarly, this work uses BG boundaries and population estimates from the United States Census Bureau’s Annual Community Survey to evaluate fine-scale inequality by race-ethnicity.^59^ While BGs provide some of the highest spatial resolution population estimates of race-ethnicity available through the Census, they do not reflect true community boundaries and can potentially yield different equity-oriented conclusions than studies that use true community boundaries.^82^ Additionally, in low population places, the Census Bureau adds model noise to avoid privacy issues, which could influence results in a small population or a specific block group. Furthermore, collecting demographic information on race and ethnicity is inherently flawed, as it relies on self-identification and reflection within a limited set of options, which can vary on an individual basis or with the exact phrasing of the question.^83,84^ These issues can be exacerbated when combined with nonresponse bias due to concerns about immigration status or other barriers (e.g., language, disability status).^85,86^

Finally, our analysis focuses only on the disparity in PM_2.5_ exposure from agricultural sources, but this is just one of many intersectional issues facing farmside communities in California. Beyond PM_2.5_, these communities are also disproportionately exposed to other environmental hazards, such as pesticides, extreme heat events, and increasingly wildfire-related PM_2.5_.^51,52,87–89^ As populations grow and climate change accelerates, there is potential for these disproportionate cumulative burdens on farm-side communities in California to increase. Although our analytical focus considers just one part of a larger, intersectional injustice for these communities, future interventions and analyses should examine the implications of these climate-driven coexposures.

### 3.4 Best Practices for Future Equity-Oriented Exposure Science

Using modeled estimates of total PM_2.5_ from agricultural activities and BG-level population data, we found that the conclusions of an equity-oriented analysis can differ based on the choices of exposure input, study geography, and reference population. The commonly utilized statewide approach at the PWM identified Hispanic Californians as the most overburdened group for emissions from the agricultural sector. This population also experiences disparate exposures along every point of their distribution curve and across all four study geographies. This modeled disparity is consistent with historical context, as those who have historically worked and settled in California’s agricultural areas have been predominantly Hispanic.^56^ By demonstrating this disparity along the distribution and across spatial extents, our analysis robustly supports the need for targeted interventions in agricultural emissions to reduce this disparate burden.

In comparison, the statewide PWM identifies Black Californians as the second most disparately exposed group, but there is substantially more nuance when considering the full distribution and varied study geographies. While a very small share of Black Californians resides in places that are highly polluted by agricultural emissions, as a group, Black Californians are not disparately exposed in the upper quartile of the statewide distribution of agricultural exposures. This is important, as evaluating disparities at the upper tails of the exposure distribution is critical for mitigating inequality in health outcomes.

Furthermore, we have shown that inappropriate selection of study geography and reference population could yield incomplete or incorrect conclusions, extending the considerations highlighted by Mohai et al.^9^ to air pollution modeling tools. Across our four example geographies, the conclusions about relative disparities for Black Californians varied. The choice of whether to calculate exposure disparities within a region or relative to a broader region also had strong implications for the magnitude of the disparities observed. Though we find mixed results about the disparity experienced by Black Californians in the aggregate, our analysis also highlights that certain subsets of this population are among the most disproportionately exposed to agricultural emissions. This is just one indication of the historical and ongoing socio-environmental injustices faced by Black people in California necessitating continued and intentional interventions to address.

The varied results we observe in terms of both magnitude and direction of disparity indicate the potential for cascading misinterpretations—both quantitative and qualitative—about exposure inequity in the absence of deliberate and clearly communicated methodological choices. This can potentially affect our overall understanding of the problem and limit how effectively policymakers can mitigate disparities. Thus, equity-oriented exposure science must critically evaluate the research question in order to determine the appropriate methodological design choices required to address it.

We recommend the following best practices for the scientific community. First, when initiating an air pollution equity analysis, a crucial first step involves critically appraising the research question and determining the exposure and disparity indicators, study geography, and reference population that are appropriate based on the decision-making and jurisdictional context for potential interventions. At this point, procedural justice considerations (e.g., engaging communities and key decision-makers in the design and scope of the proposed question and analyses) are important. Similarly, when selecting a geographic domain, it is important to evaluate who is included in that domain and how sample sizes and population patterns may affect the resulting conclusions. Second, we recommend that evaluations of equity and disparity include a demonstration of the robustness of their findings to other design choices. This could take the form of a table demonstrating how the relative disparity in exposure changes when evaluated at the mean statewide versus along a distribution or across spatial scales. Finally, clear and direct communication of the design choices that were made and the rationale behind them is crucial. Interpretable explanation of the metrics, methods, and assumptions underlying an analysis ensures that others—be they scientists, policymakers, and/or community members more broadly—understand the findings and ultimately determine how to use them to shape decisions.

These recommendations should be pursued alongside the other frameworks and best practices for quantitative exposure science described in the literature.^5–7,14,15^ For example, the framework outlined by van Horne et al.^5^ calls for systems of shared leadership, governance, participation, and ownership of scientific work with communities and stakeholders. Our work emphasizes the importance of incorporating best analytical practices in academic data science as a key additional consideration. Similarly, Gardner-Frolick et al.^14^ promote thoughtful evaluation of data and modeling tools. Once these tools are chosen, our recommendations should be applied to data analysis. Chambliss et al.^7^ call for more inclusion of intersectional approaches to exposure assessment; our findings should be leveraged to improve the derivation of the exposure-driven metrics that they discuss. Finally, we echo many of the calls for increased procedural and recognitional justice in sustainability, exposure, and implementation science raised by Giang et al.^6^ and Ashcraft et al.^15^ Our quantitative recommendations focus on the academic’s role in isolation; in practice, ethical and meaningful involvement of the impacted communities (i.e., procedural justice) may involve additional considerations on the relevant areas, communities, and pollutants studied.

Additionally, we hope that our findings and recommendations can both inform and be informed by other types of nonquantitative inquiry and research on environmental justice and exposure disparities. Quantitative answers to equity-oriented questions can provide general information about *where*, *for whom*, and *to what degree* an intervention is needed. Research techniques from the social science (e.g., participatory action research, reparative planning, community advocacy) are necessary for grounding these high-level strategies in lived experiences and community-specific contexts.^90–92^ The specific choice of study geography and reference population for understanding inequity facing a community, for example, may be meaningfully informed by interviews with community members (i.e., participatory action research). Furthermore, thoughtfully conducted quantitative analyses that identify sources of exposure disparities can guide community-led efforts to mitigate these disparities (e.g., in a reparative planning framework).^93^

While here we have focused on one case study, our findings suggest that the growing body of distributional justice-oriented literature on air pollution ought to more carefully design methods that appropriately answer outstanding questions. To be actionable, scientific work aimed to inform policy decisions needs to—at a minimum—employ the appropriate exposure and disparity metrics and study geographies to address the problem that the policy is targeting. While ultimately the important calls to monitor and track disparities rely on succinct summary statistics, standard approaches (e.g., PWM) and assumptions (e.g., nonattainment area boundary) may not always be the appropriate choices for identifying which people urgently need interventions and from what. Involving the most affected communities in some of these choices about metrics, methods, and geographic scales could both increase the rigor of the analysis and work toward greater procedural and recognitional justice.

## Supplementary Material



## Data Availability

The data and code underlying this study are available open-access on Zenodo (DOI: 10.5281/zenodo.18088533).

## Notes

Additional sensitivity analyses, supplementary figures, and a supplemental table (PDF)

## References

[1] Tessum C. W., Apte J. S., Goodkind A. L., Muller N. Z., Mullins K. A., Paolella D. A., Polasky S., Springer N. P., Thakrar S. K., Marshall J. D., Hill J. D. (2019). Inequity
in Consumption of Goods
and Services Adds to Racial−Ethnic Disparities in Air Pollution
Exposure. P. Natl. Acad. Sci. USA.

[2] Tessum C. W., Paolella D. A., Chambliss S. E., Apte J. S., Hill J. D., Marshall J. D. (2021). PM_2.5_ Polluters Disproportionately and Systemically
Affect People of Color in the United States. Sci. Adv..

[3] Lane H. M., Morello-Frosch R., Marshall J. D., Apte J. S. (2022). Historical Redlining
Is Associated with Present-Day Air Pollution Disparities in U.S. Cities. Environ. Sci. Technol. Lett..

[4] Liu J., Marshall J. D. (2023). Spatial Decomposition
of Air Pollution Concentrations
Highlights Historical Causes for Current Exposure Disparities in the
United States. Environ. Sci. Technol. Lett..

[5] Van
Horne Y. O., Alcala C. S., Peltier R. E., Quintana P. J. E., Seto E., Gonzales M., Johnston J. E., Montoya L. D., Quirós-Alcalá L., Beamer P. I. (2023). An Applied Environmental
Justice Framework for Exposure Science. J. Expo.
Sci. Env. Epid..

[6] Giang A., Edwards M. R., Fletcher S. M., Gardner-Frolick R., Gryba R., Mathias J.-D., Venier-Cambron C., Anderies J. M., Berglund E., Carley S., Erickson J. S., Grubert E., Hadjimichael A., Hill J., Mayfield E., Nock D., Pikok K. K., Saari R. K., Samudio
Lezcano M., Siddiqi A., Skerker J. B., Tessum C. W. (2024). Equity
and Modeling in Sustainability Science: Examples and Opportunities
throughout the Process. P. Natl. Acad. Sci.
USA.

[7] Chambliss S., La Frinere-Sandoval N. Q., Zigler C., Mueller E. J., Peng R. D., Hall E. M., Matsui E. C., Cubbin C. (2024). Alignment
of Air Pollution Exposure Inequality Metrics with Environmental Justice
and Equity Goals in the United States. Int.
J. Environ. Res. Pub. He..

[8] Schlosberg, D. Defining Environmental Justice: Theories, Movements, and Nature; Oxford University Press: Oxford, 2007. 10.1093/acprof:oso/9780199286294.001.0001.

[9] Mohai P., Pellow D., Roberts J. T. (2009). Environmental
Justice. Annu. Rev. Env. Resour..

[10] Koolik L. H., Bullard R. D., Min E., Morello-Frosch R., Patterson R. F., Salgado M., Wedekind N., Marshall J. D., Apte J. S. (2025). Eliminating Air Pollution Disparities
Requires More
than Emission Reduction. P. Natl. Acad. Sci.
USA.

[11] Wang Y., Apte J. S., Hill J. D., Ivey C. E., Johnson D., Min E., Morello-Frosch R., Patterson R., Robinson A. L., Tessum C. W., Marshall J. D. (2023). Air Quality Policy
Should Quantify Effects on Disparities. Science.

[12] Gohlke J. M., Harris M. H., Roy A., Thompson T. M., DePaola M., Alvarez R. A., Anenberg S. C., Apte J. S., Demetillo M. A. G., Dressel I. M., Kerr G. H., Marshall J. D., Nowlan A. E., Patterson R. F., Pusede S. E., Southerland V. A., Vogel S. A. (2023). State-of-the-Science Data and Methods Need to Guide
Place-Based Efforts to Reduce Air Pollution Inequity. Environ. Health Perspect..

[13] Mork D., Delaney S., Dominici F. (2024). Policy-Induced
Air Pollution Health
Disparities: Statistical and Data Science Considerations. Science.

[14] Gardner-Frolick R., Boyd D., Giang A. (2022). Selecting
Data Analytic and Modeling
Methods to Support Air Pollution and Environmental Justice Investigations:
A Critical Review and Guidance Framework. Environ.
Sci. Technol..

[15] Ashcraft L. E., Cabrera K. I., Lane-Fall M. B., South E. C. (2024). Leveraging Implementation
Science to Advance Environmental Justice Research and Achieve Health
Equity through Neighborhood and Policy Interventions. Annu. Rev. Publ. Health.

[16] Pratt G. C., Vadali M. L., Kvale D. L., Ellickson K. M. (2015). Traffic,
Air Pollution, Minority and Socio-Economic Status: Addressing Inequities
in Exposure and Risk. Int. J. Environ. Res.
Pub. He..

[17] Morello-Frosch R., Lopez R. (2006). The Riskscape and the Color Line: Examining the Role of Segregation
in Environmental Health Disparities. Environ.
Res..

[18] Bowe B., Xie Y., Yan Y., Al-Aly Z. (2019). Burden of
Cause-Specific Mortality
Associated With PM2.5 Air Pollution in the United States. JAMA Netw. Open.

[19] Colmer J., Hardman I., Shimshack J., Voorheis J. (2020). Disparities in PM 2.5
Air Pollution in the United States. Science.

[20] Jbaily A., Zhou X., Liu J., Lee T.-H., Kamareddine L., Verguet S., Dominici F. (2022). Air Pollution Exposure Disparities
across US Population and Income Groups. Nature.

[21] Liu J., Clark L. P., Bechle M. J., Hajat A., Kim S.-Y., Robinson A. L., Sheppard L., Szpiro A. A., Marshall J. D. (2021). Disparities
in Air Pollution Exposure in the United States by Race/Ethnicity and
Income, 1990−2010. Environ. Health Persp..

[22] Kelly J. T., Jang C., Timin B., Di Q., Schwartz J., Liu Y., van Donkelaar A., Martin R. V., Berrocal V., Bell M. L. (2021). Examining PM_2.5_ Concentrations and Exposure
Using Multiple Models. Environ. Res..

[23] Nguyen N. P., Marshall J. D. (2018). Impact, Efficiency,
Inequality, and Injustice of Urban
Air Pollution: Variability by Emission Location. Environ. Res. Lett..

[24] Levy J. I., Chemerynski S. M., Tuchmann J. L. (2006). Incorporating Concepts
of Inequality
and Inequity into Health Benefits Analysis. Int. J. Equity Health.

[25] Gosztonyi Á., Demmler J. C., Juhola S., Ala-Mantila S. (2023). Ambient Air
Pollution-Related Environmental Inequality and Environmental Dissimilarity
in Helsinki Metropolitan Area. Finland. Ecol.
Econ..

[26] Wang Y., Apte J. S., Hill J. D., Ivey C. E., Patterson R. F., Robinson A. L., Tessum C. W., Marshall J. D. (2022). Location-Specific
Strategies for Eliminating US National Racial-Ethnic PM_2.5_ Exposure Inequality. P. Natl. Acad. Sci. USA.

[27] Apte, J. S. ; Chambliss, S. E. ; Tessum, C. W. ; Marshall, J. D. A Method to Prioritize Sources for Reducing High PM_2.5_ Exposures in Environmental Justice Communities in California. Report prepared for the California Air Resources Board, 2019; pp 79. https://ww2.arb.ca.gov/sites/default/files/classic/research/apr/past/17rd006.pdf (accessed 2025-08-03).

[28] Polonik P., Ricke K., Reese S., Burney J. (2023). Air Quality Equity
in US Climate Policy. P. Natl. Acad. Sci. USA.

[29] Clark L. P., Harris M. H., Apte J. S., Marshall J. D. (2022). National and Intraurban
Air Pollution Exposure Disparity Estimates in the United States: Impact
of Data-Aggregation Spatial Scale. Environ.
Sci. Technol. Lett..

[30] Picciano P., Qiu M., Eastham S. D., Yuan M., Reilly J., Selin N. E. (2023). Air Quality
Related Equity Implications of U.S. Decarbonization Policy. Nat. Commun..

[31] de
Souza P., Anenberg S., Makarewicz C., Shirgaokar M., Duarte F., Ratti C., Durant J. L., Kinney P. L., Niemeier D. (2024). Quantifying Disparities in Air Pollution
Exposures across the United States Using Home and Work Addresses. Environ. Sci. Technol..

[32] Chambliss S. E., Pinon C. P. R., Messier K. P., LaFranchi B., Upperman C. R., Lunden M. M., Robinson A. L., Marshall J. D., Apte J. S. (2021). Local- and Regional-Scale Racial
and Ethnic Disparities
in Air Pollution Determined by Long-Term Mobile Monitoring. P. Natl. Acad. Sci. USA.

[33] Camilleri S. F., Montgomery A., Visa M. A., Schnell J. L., Adelman Z. E., Janssen M., Grubert E. A., Anenberg S. C., Horton D. E. (2023). Air Quality,
Health and Equity Implications of Electrifying Heavy-Duty Vehicles. Nat. Sustain..

[34] Koolik L. H., Alvarado Á., Budahn A., Plummer L., Marshall J. D., Apte J. S. (2024). PM_2.5_ Exposure Disparities Persist despite
Strict Vehicle Emissions Controls in California. Sci. Adv..

[35] Marshall J. D. (2008). Environmental
Inequality: Air Pollution Exposures in California’s South Coast
Air Basin. Atmos. Environ..

[36] Torbatian S., Saleh M., Xu J., Minet L., Gamage S. M., Yazgi D., Yamanouchi S., Roorda M. J., Hatzopoulou M. (2024). Societal Co-Benefits
of Zero-Emission Vehicles in the Freight Industry. Environ. Sci. Technol..

[37] McNeil W. H., Porzio J., Tong F., Harley R. A., Auffhammer M., Scown C. D. (2025). Impact of Truck
Electrification on Air Pollution Disparities
in the United States. Nat. Sustain..

[38] Goforth T., Nock D. (2022). Air Pollution Disparities
and Equality Assessments of US National
Decarbonization Strategies. Nat. Commun..

[39] Harper S., King N., Meersman S. C., Reichman M. E., Breen N., Lynch J. (2010). Implicit Value Judgments
in the Measurement of Health Inequalities. Milbank
Q..

[40] Levy J. I., Greco S. L., Melly S. J., Mukhi N. (2009). Evaluating Efficiency-Equality
Tradeoffs for Mobile Source Control Strategies in an Urban Area. Risk Anal..

[41] Buckley L., Arter C. A., Willis M. D., Geddes J. A., Rick C., Kinney P. L., Arunachalam S., Buonocore J. J., Levy J. I. (2024). A Comparison of Population-Level
Exposure and Equity
Tradeoffs across Strategies to Reduce Fine Particulate Matter Emissions
from Transportation Sources in the Northeastern US. Environ. Res..

[94] Marshall, J. D. ; Koolik, L. H. ; Unal, A. ; Morello-Frosch, R. ; Apte, J. S. Advancing Methods and Models that Promote Equity in Ambient Air Quality. Annu. Rev. Public Health 2025, 47, 10.1146/annurev-publhealth-091824-125106. 41417974

[42] Baden B. M., Noonan D. S., Turaga R. M. R. (2007). Scales of Justice: Is There a Geographic
Bias in Environmental Equity Analysis?. J. Environ.
Plann. Man..

[43] Carvalho C., Del Campo A. G., de Carvalho Cabral D. (2022). Scales of
Inequality: The Role of
Spatial Extent in Environmental Justice Analysis. Landscape Urban Plan..

[44] GBD
Risk Factor Collaborators (2020). Global Burden of 87 Risk Factors in 204 Countries and Territories,
1990−2019: A Systematic Analysis for the Global Burden of Disease
Study 2019. Lancet.

[45] Marshall J. D., Apte J. S., Coggins J. S., Goodkind A. L. (2015). Blue Skies Bluer?. Environ. Sci.
Technol..

[46] Pope C. A., Cropper M., Coggins J., Cohen A. (2015). Health Benefits
of Air Pollution Abatement Policy: Role of the Shape of the Concentration−Response
Function. J. Air Waste Manage..

[47] Geldsetzer P., Fridljand D., Kiang M. V., Bendavid E., Heft-Neal S., Burke M., Thieme A. H., Benmarhnia T. (2024). Disparities
in Air Pollution Attributable Mortality in the US Population by Race/Ethnicity
and Sociodemographic Factors. Nat. Med..

[48] Josey K. P., Delaney S. W., Wu X., Nethery R. C., DeSouza P., Braun D., Dominici F. (2023). Air Pollution
and Mortality at the
Intersection of Race and Social Class. N. Engl.
J. Med..

[49] Domingo N. G. G., Balasubramanian S., Thakrar S. K., Clark M. A., Adams P. J., Marshall J. D., Muller N. Z., Pandis S. N., Polasky S., Robinson A. L., Tessum C. W., Tilman D., Tschofen P., Hill J. D. (2021). Air Quality−Related Health
Damages of Food. P. Natl. Acad. Sci. USA.

[50] Sun P., Farley R. N., Li L., Srivastava D., Niedek C. R., Li J., Wang N., Cappa C. D., Pusede S. E., Yu Z., Croteau P., Zhang Q. (2022). PM_2.5_ Composition and Sources in the San Joaquin Valley of California:
A Long-Term Study Using ToF-ACSM with the Capture Vaporizer. Environ. Pollut..

[51] Castillo F., Mora A. M., Kayser G. L., Vanos J., Hyland C., Yang A. R., Eskenazi B. (2021). Environmental Health
Threats to Latino
Migrant Farmworkers. Annu. Rev. Publ. Health.

[52] Cheney A. M., Barrera T., Rodriguez K., Jaramillo López A. M. (2022). The Intersection
of Workplace and Environmental Exposure on Health in Latinx Farm Working
Communities in Rural Inland Southern California. Int. J. Environ. Res. Pub. He..

[53] Hill A. E., Burkhardt J., Bayham J., O’Dell K., Ford B., Fischer E. V., Pierce J. R. (2024). Air Pollution, Weather,
and Agricultural Worker Productivity. Am. J.
Agr. Econ..

[54] Matias S. L., French C. D., Gomez-Lara A., Schenker M. B. (2022). Chronic Disease
Burden among Latino Farmworkers in California. Front. Public Health.

[55] Yearby R., Lewis C., Gibson C. (2023). Incorporating Structural Racism,
Employment Discrimination, and Economic Inequities in the Social Determinants
of Health Framework to Understand Agricultural Worker Health Inequities. Am. J. Public Health.

[56] Allensworth E. M., Rochín R. I. (1998). Ethnic
Transformation in Rural California: Looking
Beyond the Immigrant Farmworker. Rural Sociol..

[57] United States EPA . 2014 National Emissions Inventory, 2018. https://www.epa.gov/air-emissions-inventories/2014-national-emissions-inventory-nei-data#doc (accessed 2025-08-03).

[58] Tessum C.
W., Hill J. D., Marshall J. D. (2017). InMAP: A Model for Air Pollution
Interventions. PLoS One.

[59] Manson S., Schroeder J., Van Riper D., Knowles K., Kugler T., Roberts F., Ruggles S. (2024). IPUMS National Historical Geographic
Information System: Version 19.0. IPUMS.

[60] California Department of Conservation . California Important Farmland 2014, 2016. https://gis.conservation.ca.gov/portal/home/item.html?id=be4d1aff89824309b9300f4fdd3c64f2 (accessed 2025-08-03).

[61] California Department of Pesticide Regulation . Pesticides Use Report 2014, 2022. https://calpip.cdpr.ca.gov/infodocs.cfm (accessed 2025-08-03).

[62] California Department of Water Resources . Statewide Crop Mapping 2014. California Natural Resources Agency Open Data, 2023. https://data.cnra.ca.gov/dataset/statewide-crop-mapping (accessed 2025-08-03).

[63] van
Donkelaar A., Hammer M. S., Bindle L., Brauer M., Brook J. R., Garay M. J., Hsu N. C., Kalashnikova O. V., Kahn R. A., Lee C., Levy R. C., Lyapustin A., Sayer A. M., Martin R. V. (2021). Monthly Global Estimates of Fine
Particulate Matter and Their Uncertainty. Environ.
Sci. Technol..

[64] Kim S.-Y., Bechle M., Hankey S., Sheppard L., Szpiro A. A., Marshall J. D. (2020). Concentrations of Criteria Pollutants in the Contiguous
U.S., 1979 − 2015: Role of Prediction Model Parsimony in Integrated
Empirical Geographic Regression. PLoS One.

[65] Marshall J. D., Riley W. J., McKone T. E., Nazaroff W. W. (2003). Intake
Fraction
of Primary Pollutants: Motor Vehicle Emissions in the South Coast
Air Basin. Atmos. Environ..

[66] Graves, S. Racial Disparities in California’s State Prisons Remain Large Despite Justice System Reforms; California Budget & Policy Center, 2021. https://calbudgetcenter.org/app/uploads/2021/06/R-FP-Prison-Racial-Disparities.pdf (accessed 2025-10-01).

[67] Massoglia M., Pridemore W. A. (2015). Incarceration
and Health. Annu.
Rev. Sociol..

[68] Wildeman C., Wang E. A. (2017). Mass Incarceration, Public Health, and Widening Inequality
in the USA. Lancet.

[69] Ovienmhada U., Diongue A., Pellow D. N., Wood D. (2024). Satellite Remote Sensing
for Environmental Data Justice: Perspectives from Anti-Prison Community
Organizers on the Uses of Geospatial Data. Environ.
Justice.

[70] Pellow D. N. (2021). Struggles
for Environmental Justice in US Prisons and Jails. Antipode.

[71] Pellow, D. N. ; Lake, F. R. ; Wilson, C. A. ; Baker, E. J. Environmental Justice Struggles in Prisons and Jails Around the World: The 2020 Annual Report of the Prison Environmental Justice Project; Global Environmental Justice Project: UC Santa Barbara, 2020. https://gejp.es.ucsb.edu/sites/default/files/sitefiles/publication/Prison%20EJ%20Project%202020%20Report-compressed.pdf (accessed 2025-10-01).

[72] The Toxic Prisons Mapping Project. https://www.toxicprisons.com/ (accessed 2025-10-01).

[73] Novotny E. V., Bechle M. J., Millet D. B., Marshall J. D. (2011). National
Satellite-Based
Land-Use Regression: NO_2_ in the United States. Environ. Sci. Technol..

[74] Bechle M. J., Millet D. B., Marshall J. D. (2013). Remote
Sensing of Exposure to NO_2_: Satellite Versus Ground-Based
Measurement in a Large Urban
Area. Atmos. Environ..

[75] Clark L. P., Millet D. B., Marshall J. D. (2014). National
Patterns in Environmental
Injustice and Inequality: Outdoor NO_2_ Air Pollution in
the United States. PLoS One.

[76] Qi M., Dixit K., Marshall J. D., Zhang W., Hankey S. (2022). National Land
Use Regression Model for NO_2_ Using Street View Imagery
and Satellite Observations. Environ. Sci. Technol..

[77] Ma Y., Zang E., Opara I., Lu Y., Krumholz H. M., Chen K. (2023). Racial/Ethnic
Disparities in PM_2.5_-Attributable Cardiovascular
Mortality Burden in the United States. Nat.
Hum. Behav..

[78] Spiller E., Proville J., Roy A., Muller N. Z. (2021). Mortality
Risk from
PM_2.5_: A Comparison of Modeling Approaches to Identify
Disparities across Racial/Ethnic Groups in Policy Outcomes. Environ. Health Persp..

[79] Zimmerman F. J., Anderson N. W. (2019). Trends in Health Equity in the United States by Race/Ethnicity,
Sex, and Income, 1993−2017. JAMA Netw.
Open.

[80] Liévanos R. S. (2015). Race, Deprivation,
and Immigrant Isolation: The Spatial Demography of Air-Toxic Clusters
in the Continental United States. Soc. Sci.
Res..

[81] Rubio R., Grineski S., Collins T. (2021). Children’s Exposure to Vehicular
Air Pollution in the United States: Environmental Injustices at the
Intersection of Race/Ethnicity and Language. Environ. Sociol..

[82] Williams R. W. (1999). The Contested
Terrain of Environmental Justice Research: Community as Unit of Analysis. Soc. Sci. J..

[83] Strmic-Pawl H. V., Jackson B. A., Garner S. (2018). Race Counts: Racial
and Ethnic Data
on the U.S. Census and the Implications for Tracking Inequality. Sociol. Race Ethnicity.

[84] Prewitt K. (2018). The Census
Race Classification: Is It Doing Its Job?. Ann.
Am. Acad. Polit. SS..

[85] Kissam E. (2019). How Low Response
among Latino Immigrants Will Lead to Differential Undercount If the
United States’ 2020 Census Includes a Question on Sensitive
Citizenship. Stat. J. IAOS.

[86] Quiros S. M., O’Hare W. P. (2024). Towards
a More Accurate Count: Identifying State-Level
Predictors of the Undercount of Young Hispanic Children in the 2010
and 2020 U.S. Censuses. Popul. Res. Policy Rev..

[87] Chunga
Pizarro C. A., Buchholz R. R., Hornbrook R. S., Christensen K., Méndez M. (2024). Air Quality Monitoring and the Safety
of Farmworkers in Wildfire Mandatory Evacuation Zones. GeoHealth.

[88] Kamai E. M., Ruiz B. C., Van Horne Y. O., Barahona D. D., Bejarano E., Olmedo L., Eckel S. P., Johnston J. E., Farzan S. F. (2023). Agricultural
Burning in Imperial Valley, California and Respiratory Symptoms in
Children: A Cross-Sectional, Repeated Measures Analysis. Sci. Total Environ..

[89] Schollaert C., Connolly R., Cushing L., Jerrett M., Liu T., Marlier M. (2025). Air Quality Impacts
of the January 2025 Los Angeles
Wildfires: Insights from Public Data Sources. Environ. Sci. Technol. Lett..

[90] Bowness E., Tremembé M., Tremembé L., Azevedo A., Tavares F. C., Pasek A., Ahenakew C., Valley W., Stein S. (2025). Beyond Redistribution:
A Framework for Reparative Just Transitions. Environ. Res. Lett..

[91] Camponeschi C. (2022). Toward Integrative
Resilience: A Healing Justice and Trauma-Informed Approach to Urban
Climate Planning. Cities Health.

[92] Commodore A., Wilson S., Muhammad O., Svendsen E., Pearce J. (2017). Community-Based
Participatory Research for the Study of Air Pollution: A Review of
Motivations, Approaches, and Outcomes. Environ.
Monit. Assess..

[93] Rick C., Gaddy K., Lewis S., Mitchell M., Owen S., Shabazz Q., Chu-Wiens L., Stange J., Little C., Ellis E., Arter C., Kinney P., Levy J. I., Perera F., Coomes K., Lau K., Buckley L., Raifman M., C D., Arunachalam S., Buonocore J. (2024). Modeling Air Pollution-Related Health Benefits of Transportation
Scenarios: A Collaboration Between Academic Researchers and Environmental
Justice Organizations. Community Sci..

