# Supplementary material for: Methodological Design Choices Can Affect Air Pollution Exposure Disparity Estimates: A Case Study on California’s Agricultural Sector
Source: Environ Sci Technol. 2026 Feb 5;60(6):4753–63. doi: 10.1021/acs.est.5c10796 (PMC12918526; doi:10.1021/acs.est.5c10796)
Supplement: Supplementary file 1 [file es5c10796_si_001.pdf]

Supporting Information for

Methodological Design Choices Can Affect Air Pollution Exposure Disparity Estimates: A Case Study on California's Agricultural Sector

Libby H. Koolik[1], Simone Speizer[2], Clara Rong[1,3], Sarah Chambliss[4], Julian D. Marshall[5], Rachel Morello-Frosch[6,7], Christopher W. Tessum[8], Joshua S. Apte[1,7]*

[1] Department of Civil and Environmental Engineering; University of California, Berkeley; Berkeley, CA, USA
[2] Energy and Resources Group; University of California, Berkeley; Berkeley, CA, USA
[3] Department of Civil and Environmental Engineering; Massachusetts Institute of Technology; Cambridge, MA, USA
[4] Department of Population Health; University of Texas at Austin Dell Medical School; Austin, TX, USA
[5] Department of Civil and Environmental Engineering; University of Washington; Seattle, WA, USA
[6] Department of Environmental Science, Policy, and Management; University of California, Berkeley; Berkeley, CA, USA
[7] School of Public Health; University of California, Berkeley; Berkeley, CA, USA
[8] Department of Civil and Environmental Engineering; University of Illinois at Urbana−Champaign, Urbana, IL, USA

*Corresponding author: Joshua S. Apte (email: apte@berkeley.edu)

This PDF file includes 15 pages containing:

1. Supporting Software and Datasets
2. Figures S1 to S8
3. Table S1
4. SI References

Supporting Software and Datasets

All geospatial and numerical analysis is conducted in Python (version 3.9.21), using open-source geospatial and data science packages. Key packages used included numpy[1] (version 1.24.2), pandas[2] (version 1.1.5), Fiona[3] (version 1.8.21), scipy[4] (version 1.13.1), pyarrow[5] (version 21.0.0), netCDF4[6] (version 1.7.2), and geopandas[7] (version 0.9.0) for data analysis, and seaborn[8] (version 0.12.2), matplotlib[9] (version 3.7.1), and cmcrameri[10] (version 0.12) for plotting. We also used the shapefile provided by the California Air Resources Board to define the San Joaquin Valley Air District boundary.[11] All code and data are available on Zenodo (DOI: 10.5281/zenodo.18088533).

Supporting Figures

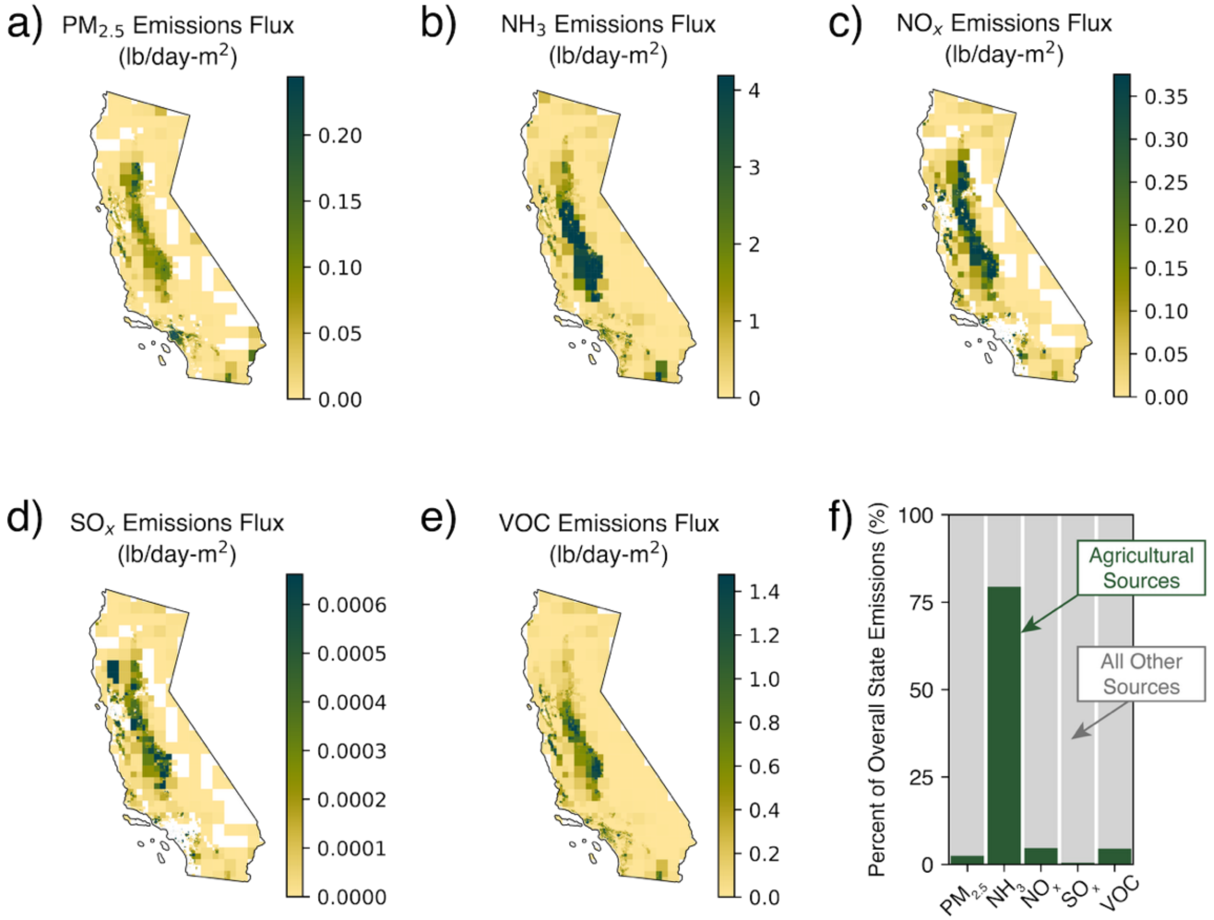

Figure S1. Emissions from the agricultural sector in California. Gridded area-normalized emission fluxes (units: lb/day-m^2) of (a) primary $PM_{2.5}$, (b) NH_3, (c) NO_x, (d) SO_x, and (e) VOC from the 2014 National Emissions inventory for the agricultural sector are plotted over the Californian domain by pollutant. Agricultural emissions are mostly concentrated in California's San Joaquin Valley. Secondary emission flux hotspots are located in northern California (near wine-growing regions) as well as coastal agricultural lands in central and southern California. (f) The relative contribution of agriculture to California's annual emissions are shown by pollutant, highlighting the importance of the agricultural sector for California's overall NH_3 emissions.

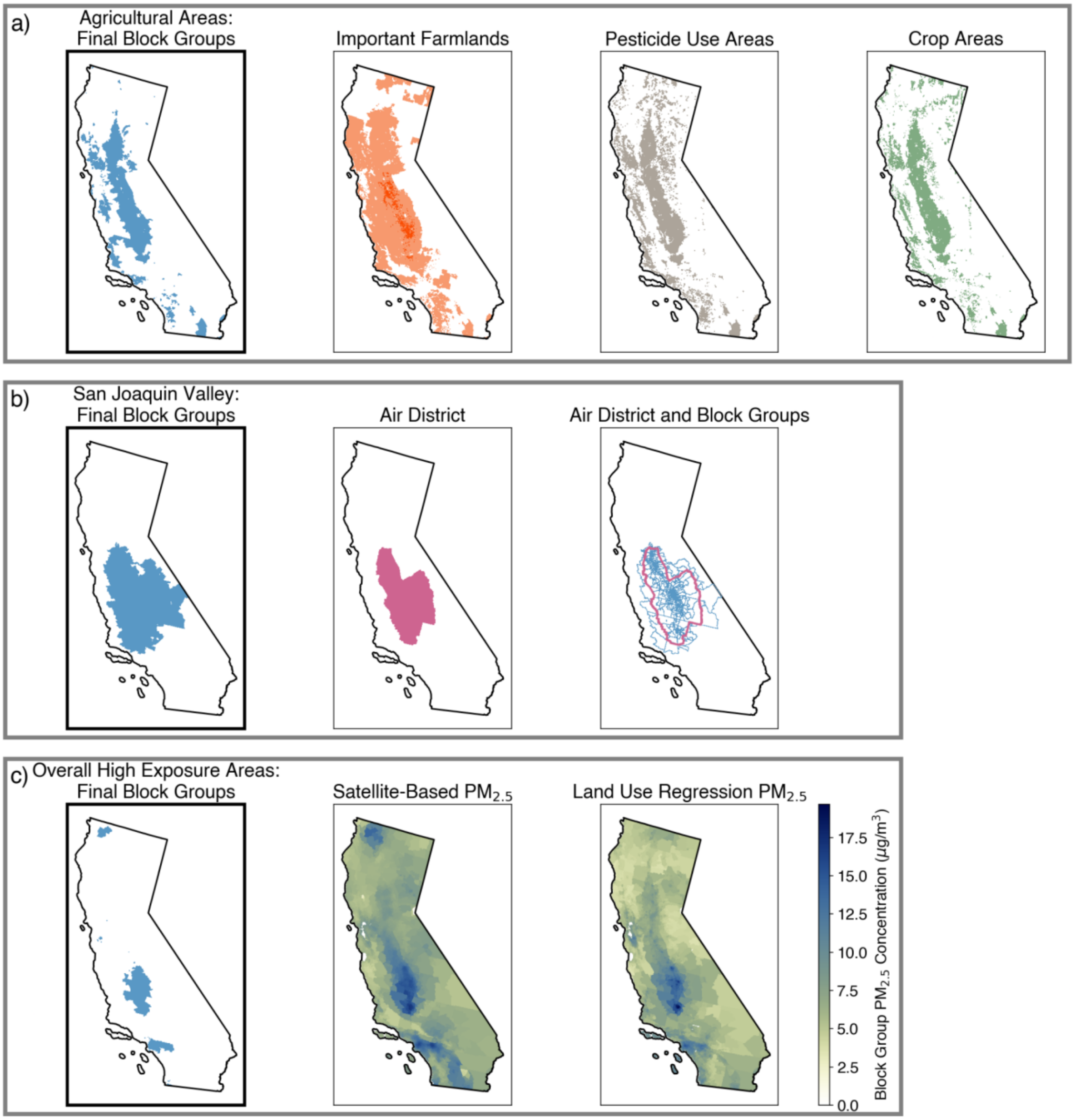

Figure S2. Study geographies used in this analysis. The first column shows the final block groups included in each of the smaller-than-statewide domains: (a) Agricultural Areas, (b) the San Joaquin Valley, and (c) Overall High Exposure Areas. Subsequent columns provide more detail about the input data used to define each of these domains. All three domains are constructed by spatially joining one or more external geospatial datasets to California's Census block groups. In (a), the Agricultural Areas are constructed based on three datasets: the California Department of Conservation's Important Farmlands dataset[12], Pesticide Use Report data from the California Department of Pesticide Regulation[13], and the California Department of Water Resources's Statewide Crop Mapping dataset[14]. From the Important Farmlands dataset, we include land in the categories of Prime Farmland, Farmland of Statewide Importance, Unique Farmland, Farmland of Local Importance, Grazing Land, Confined Animal Agriculture, and Semi-Agricultural and Rural Commercial Land. The Confined Animal Agriculture areas

are highlighted on this map in a darker orange color. For the pesticides use data, we only include production agriculture areas with a nonzero amount of land treated with pesticides, and where the area treated is reported in acres or square feet. From the crop mapping dataset, we exclude greenhouse activities, managed wetlands, idle crop land, and miscellaneous grasses. The resulting land areas from each of these three datasets are shown in columns 2-4. We find the intersection of each of these three datasets with all California block groups and identify, for each dataset, the subset of block groups for which at least 25% of their land area intersects with that dataset. If a block group meets this criteria for at least two of the three datasets, we define it as "agricultural," with the resulting Agricultural Areas block groups shown in column 1. To ensure we are not dropping confined animal agriculture (which may not be part of the pesticide or crop datasets), we have shaded the confined animal agriculture layer within the Important Farmland dataset. In (b), we include all block groups with any intersection with the San Joaquin Valley Air District in our definition of the San Joaquin Valley, shown in column 1. Column 2 shows the San Joaquin Valley Air District, while column 3 shows the boundaries of the Air District (pink) and the block groups that it intersects with (blue). In (c), Overall High Exposure Areas include block groups that are at or above the 75th percentile of total population-weighted exposure for 2014, as determined by satellite-based or empirically modeled estimates of total $PM_{2.5}$ (shown in columns 2 and 3, respectively).[11,12]

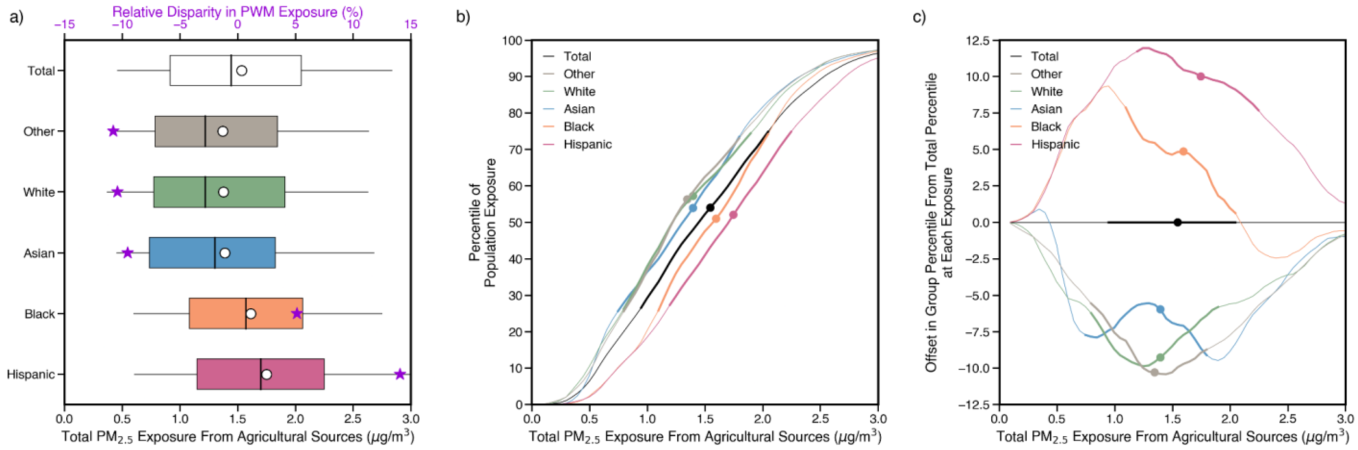

Figure S3. Distributions of $PM_{2.5}$ concentrations across California by race-ethnicity provide a more nuanced understanding of disparities than a simple mean. Here, we show the same results as in **Fig. 2** in the main text, but for all racial-ethnic groups. (a) Box plots demonstrate the range of block group exposures by race-ethnicity compared to the total population. The symbols on the box plot per group are as follows: the population-weighted mean is a circle, the median is a bar, the interquartile range is the box, and the 5th / 95th percentile are the whiskers. Superimposed on each box is a purple star representing the relative disparity in exposure estimated at the population-weighted mean (corresponding to the top x-axis). (b) We show distribution curves for binned $PM_{2.5}$ exposures (bin size = 0.05 $\mu g/m^3$), where we take the mean of the percentile values corresponding to exposures in each bin. The thicker lines represent exposures between the interquartile range for each group. We truncate the figure at 3 $\mu g/m^3$, just above the 96th percentile exposure for the total population. Circle markers indicate the population-weighted means. (c) Each group's exposure distribution curve is transformed into an offset to demonstrate how absolute exposure disparities evolve across the distribution. The offset represents how much earlier in the percentile distribution a given $PM_{2.5}$ exposure occurs for that group relative to the total population (i.e., for a given $PM_{2.5}$ concentration, the offset is the difference between the total population percentile at that concentration and the group's percentile at that concentration).

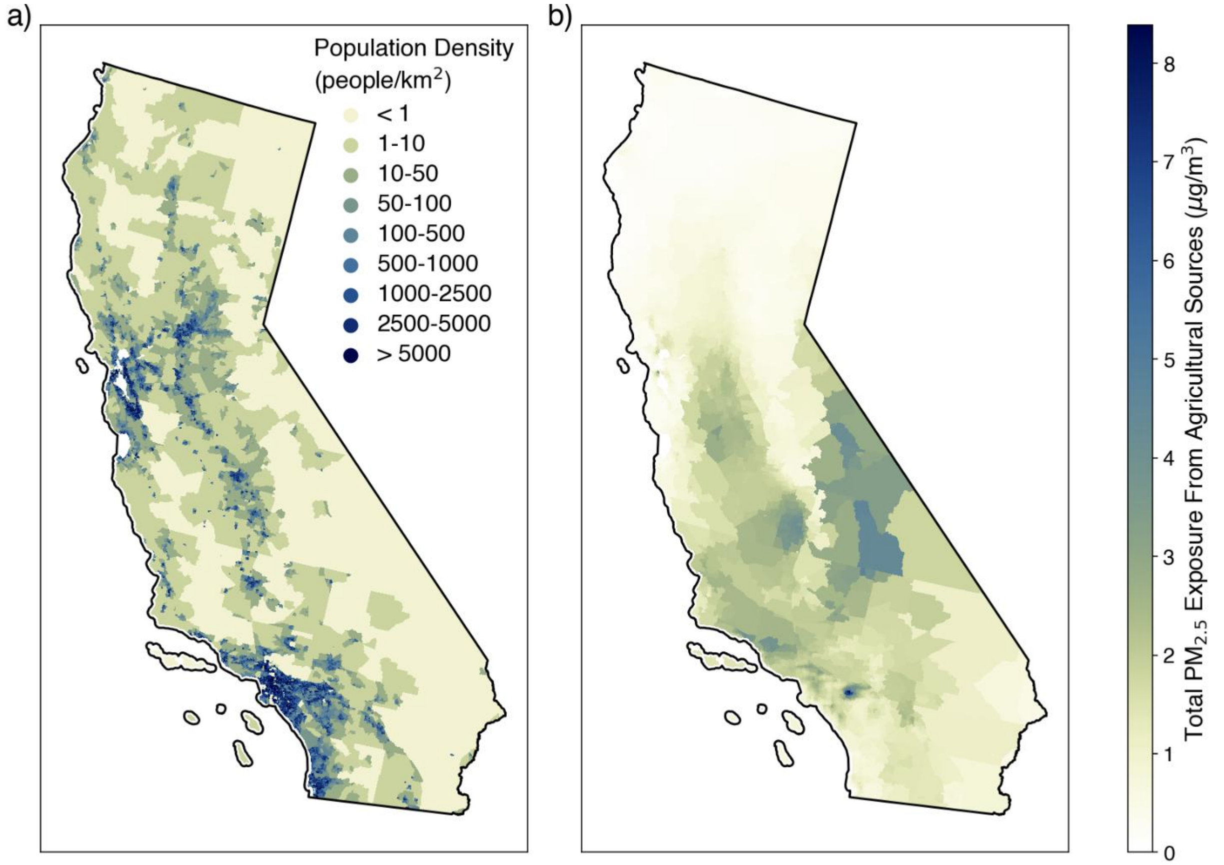

Figure S4. Modeled concentration and population distribution across California. Here, we demonstrate the results of the concentration modeling and the demographic data in space. (a) The total population density of each block group (n=23,192) in California is shown, indicating population hotspots. (b) The $PM_{2.5}$ concentrations are shown for each block group, indicating exposure hotspots.

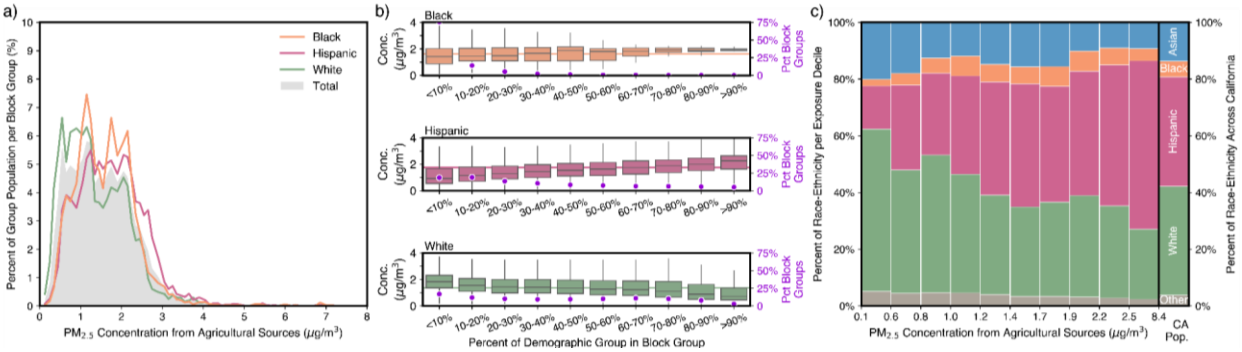

Figure S5. Three approaches to understanding patterns in exposure and demographic composition. Block group distributions of exposure and population characteristics are compared using three different techniques. All three approaches highlight the positive relationship between where Hispanic people reside and where block group exposures are higher. (a) In the first approach, we estimate the fraction of each demographic group residing at each concentration level. Block groups are sorted by concentration and binned into 75 equally-spaced concentration bins. The population of each group is then summed and compared to the overall statewide population of that group. Here, we demonstrate that the Hispanic population is more evenly distributed across higher concentration block groups than the Black or White populations. (b) In the second approach, we demonstrate how concentration distributions vary as a function of demographics. We estimate the range of concentrations for block groups with specific demographic characteristics as follows. For all block groups, we estimate the percent of each demographic group. We then bin block groups based on the percent of each demographic group. The boxes represent the interquartile ranges of concentrations in each bin; the line and fliers represent the median and 5th/95th percentiles, respectively. We also show the percent of total block groups represented by each box. Note that the Black population in California is substantially smaller than the Hispanic or White populations (5.8% Black, as compared to 38% Hispanic and White). The boxes indicate that with an increased proportion of Hispanic residents, there is an increased median concentration. (c) In the third approach, we estimate the demographic differences for block groups based on the total population exposure distribution. We bin block groups based on ten equal-population bins derived from the total population distribution. Within each bin, we calculate the racial-ethnic composition of people residing in those block groups. The final column represents the total California population for comparison. Here, we see that more polluted regions are disproportionately composed of Hispanic residents.

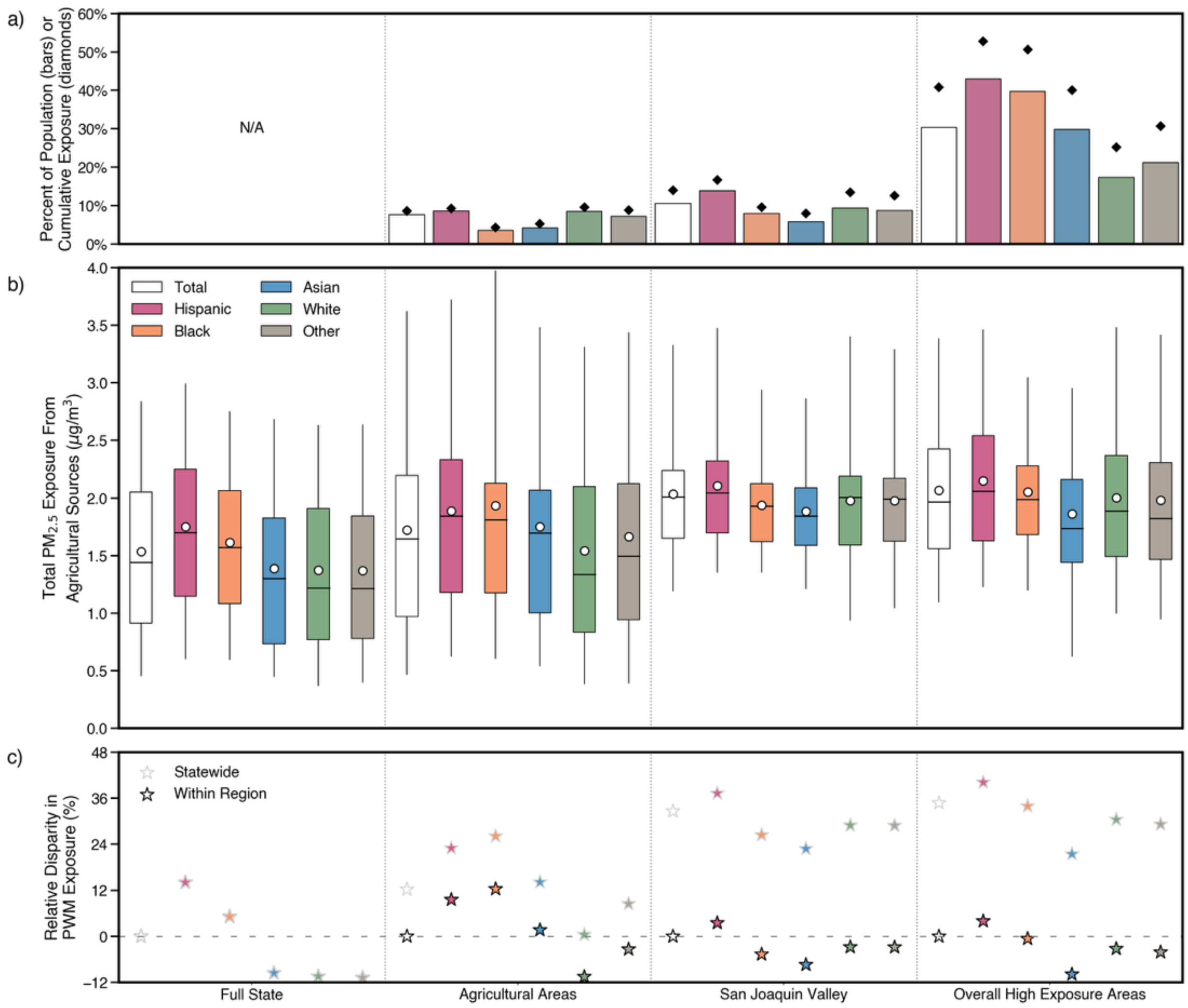

Figure S6. Exposure to $PM_{2.5}$ from agricultural sources for all racial-ethnic groups across the four study geographies. We repeat the analysis from **Fig. 3** on all racial-ethnic groups included in **Fig. S3**. The two main conclusions from Fig. 3 are reinforced with the inclusion of all groups. First, as with **Fig. 3**, depending on the study geography, the relative ordering of most to least disparately exposed groups varies. For example, across the full state and in Agricultural Areas, White Californians are the least exposed to $PM_{2.5}$ from agricultural sources at the PWM. In the San Joaquin Valley and the Overall High Exposure Areas, the Asian population is the least exposed. Second, we see that in the subgeographies with higher overall exposure to emissions from the agricultural sector, the inter-group relative disparities are less than the disparities when compared to the full state population. Notably, even the groups that are below their regional PWM are above the statewide PWM.

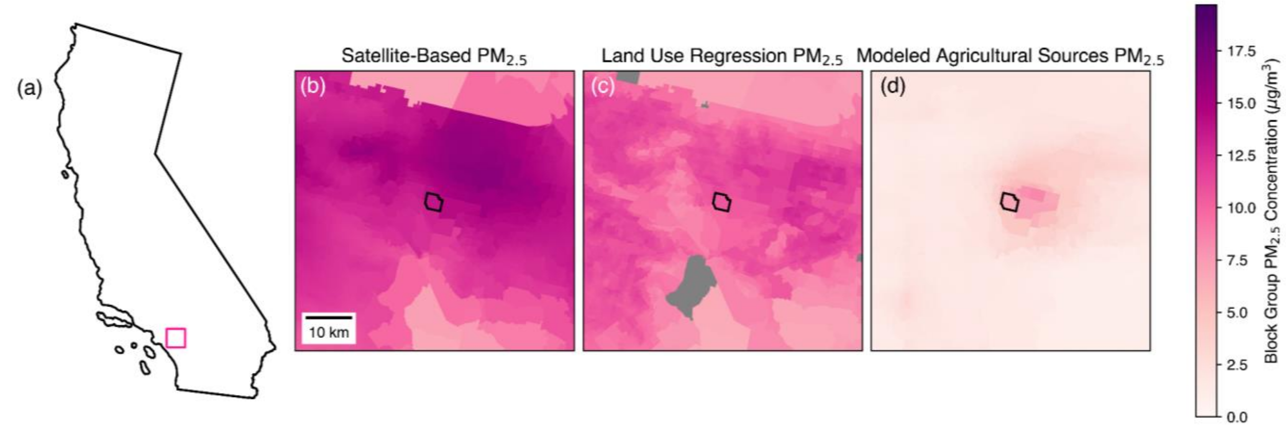

Figure S7. Fine particulate matter exposure surrounding the block group containing the California Institution for Men. Here, we zoom in on the ~60 km surrounding the California Institution for Men in Chino, CA. (a) The extent of the inset box is depicted atop California to provide spatial context. In (b) and (c), we demonstrate the relatively high total $PM_{2.5}$ concentrations from all sources surrounding this facility using two observationally-constrained datasets.[15,16] The block group containing the prison is outlined in black. Block groups with no data are depicted in gray. (d) The results of our modeled estimates for exposure to $PM_{2.5}$ from agricultural sources are visualized in the area immediately surrounding the facility. All three datasets show concentration hotspots around the prison. The block group containing the prison is not the block group with the highest exposure from agricultural sources (d); however, the relatively large number of Black individuals residing within this block group results in an outsized impact on the Black population's exposure distribution.

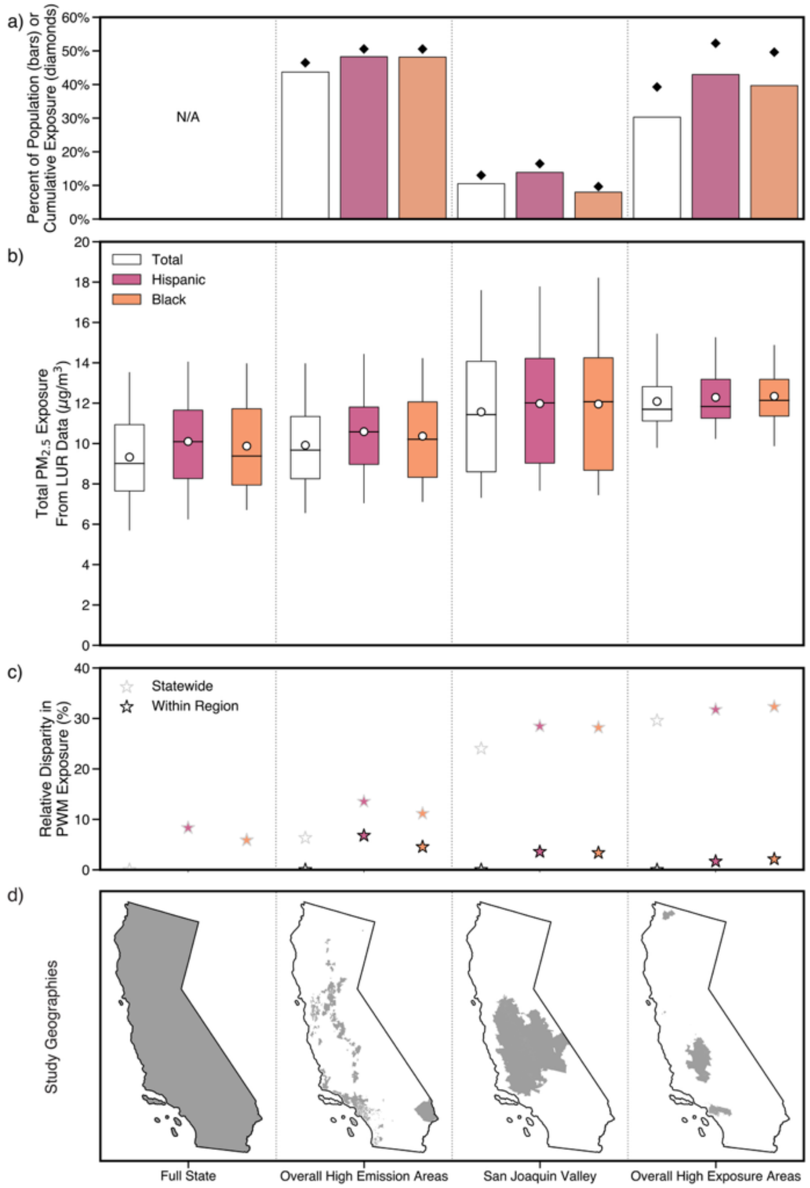

Figure S8. Exposure and disparity for Hispanic and Black Californians using four different geographic domains on observationally-constrained data. Here, we repeat the analysis in **Fig. 3** using observationally-constrained, spatially complete estimates of total economy-wide $PM_{2.5}$ from an empirically-modeled dataset.[15] Overall, we find that the same general patterns and conclusions are upheld. Depending on the study geography, the most disparately exposed group can vary. Additionally, using the full statewide exposure as the reference population results in substantially higher disparity. (a) The percent of the population present in each study geography and the cumulative exposure is shown. (b) The distributions of exposures for each group are shown. Note that the range of exposure concentrations from the full observationally-constrained data is much higher than for the agricultural emissions-alone case in **Fig. 3**. (c) The relative disparity in exposure at the population-weighted mean once again demonstrates that the relative disparity is substantially higher when calculated using a comparison to the statewide population versus the total population within each study geography. (d) For context, the block groups considered in each study geography are shaded in gray.

Supporting Table

Table S1. Comparison of disparity calculated with different choices of reference populations. We report disparity calculated at the population-weighted mean (PWM) $PM_{2.5}$ exposure for each racial-ethnic group in each region, relative to four different comparison groups: 1) the total population within that region, 2) the least exposed group within that region, 3) the total population statewide, and 4) the least exposed group statewide. Note that while we report PWMs rounded to the tenths place in this table, we calculate the absolute and relative disparities using the non-rounded PWMs, and then round these disparity estimates for reporting. These results highlight how choosing a different comparison group can dramatically change the magnitude of disparities observed. For example, in the San Joaquin Valley, the white population is less exposed on average compared to the total population within that region. However, the average white resident of the San Joaquin Valley experiences a 29% higher exposure (+0.4 µg/m^3) than the total statewide population, and a 45% higher exposure (+0.6 µg/m^3) than the least exposed group statewide.

Region	Racial-ethnic group	PWM $PM_{2.5}$ exposure (µg/m^3)	Absolute disparity at the PWM (µg/m^3)				Relative disparity at the PWM (%)			
			Compared to total population within the region	Compared to least exposed group within the region	Compared to total population statewide	Compared to least exposed group statewide	Compared to total population within the region	Compared to least exposed group within the region	Compared to total population statewide	Compared to least exposed group statewide
Full state	Total	1.5	–	0.2	–	–	–	12	–	–
	Asian	1.4	-0.1	0	–	–	-10	1	–	–
	Black	1.6	0.1	0.2	–	–	5	18	–	–
	Hispanic	1.7	0.2	0.4	–	–	14	28	–	–
	White	1.4	-0.2	0	–	–	-10	0	–	–
	Other	1.4	-0.2	–	–	–	-11	0	–	–
Agricultural Areas	Total	1.7	–	0.2	0.2	0.4	–	12	12	26
	Asian	1.8	0	0.2	0.2	0.4	2	14	14	28
	Black	1.9	0.2	0.4	0.4	0.6	12	26	26	41
	Hispanic	1.9	0.2	0.3	0.4	0.5	10	22	23	38
	White	1.5	-0.2	–	0	0.2	-10	0	0	13
	Other	1.7	-0.1	0.1	0.1	0.3	-3	8	9	22

Region	Racial-ethnic group	PWM $PM_{2.5}$ exposure (µg/m^3)	Absolute disparity at the PWM (µg/m^3)				Relative disparity at the PWM (%)			
			Compared to total population within the region	Compared to least exposed group within the region	Compared to total population statewide	Compared to least exposed group statewide	Compared to total population within the region	Compared to least exposed group within the region	Compared to total population statewide	Compared to least exposed group statewide
San Joaquin Valley	Total	2.0	–	0.2	0.5	0.7	–	8	33	49
	Asian	1.9	-0.2	–	0.4	0.5	-7	0	23	38
	Black	1.9	-0.1	0.1	0.4	0.6	-5	3	26	42
	Hispanic	2.1	0.1	0.2	0.6	0.7	4	12	37	54
	White	2.0	-0.1	0.1	0.4	0.6	-3	5	29	45
	Other	2.0	-0.1	0.1	0.4	0.6	-3	5	29	44
Overall High Exposure Areas	Total	2.1	–	0.2	0.5	0.7	–	11	35	51
	Asian	1.9	-0.2	–	0.3	0.5	-10	0	21	36
	Black	2.1	0	0.2	0.5	0.7	-1	10	34	50
	Hispanic	2.1	0.1	0.3	0.6	0.8	4	15	40	57
	White	2.0	-0.1	0.1	0.5	0.6	-3	7	30	46
	Other	2.0	-0.1	0.1	0.4	0.6	-4	6	29	45

168

SI References

(1) Harris, C. R.; Millman, K. J.; van der Walt, S. J.; Gommers, R.; Virtanen, P.; Cournapeau, D.; Wieser, E.; Taylor, J.; Berg, S.; Smith, N. J.; Kern, R.; Picus, M.; Hoyer, S.; van Kerkwijk, M. H.; Brett, M.; Haldane, A.; Fernández del Río, J.; Wiebe, M.; Peterson, P.; Gérard-Marchant, P.; Sheppard, K.; Reddy, T.; Weckesser, W.; Abbasi, H.; Gohlke, C.; Oliphant, T. E. Array Programming with NumPy. *Nature* **2020**, *585*, 357–362. DOI: 10.1038/s41586-020-2649-2.

(2) Reback, J.; McKinney, W.; jbrockmendel; Van den Bossche, J.; Augspurger, T.; Cloud, P.; gfyoung; Sinhrks; Hawkins, S.; Roeschke, M.; Klein, A.; Petersen, T.; Tratner, J.; She, C.; Ayd, W.; Naveh, S.; Garcia, M.; Schendel, J.; Hayden, A.; Saxton, D.; Jancauskas, V.; McMaster, A.; Battiston, P.; Seabold, S.; patrick; Dong, K.; chris-b1; h-vetinari; Hoyer, S.; Gorelli, M. pandas-dev/pandas: Pandas 1.1.5 (v1.1.5). **2020**. *Zenodo*. DOI: 10.5281/zenodo.4309786.

(3) Gillies, S.; Buffat, R.; Arnott, J.; Taves, M. W.; Wurster, K.; Snow, A. D.; Cochran, M.; Sales de Andrade, E; Perry, M. Fiona (v. 1.8.21). **2022**. *Github*. Available at: https://github.com/Toblerity/Fiona/tree/main.

(4) Virtanen, P.; Gommers, R.; Oliphant, T. E.; Haberland, M.; Reddy, T.; Cournapeau, D.; Burovski, W.; Peterson, P.; Weckesser, W.; Bright, J.; van der Walt, S. J.; Brett, M.; Wilson, J.; Millman, K. J.; Mayorov, N.; Nelson, A. R. J.; Jones, E.; Kern, R.; Larson, E.; Carey, C. J. Polat, I.; Feng, Y.; Moore, E. W.; VanderPlas, J.; Laxalde, D.; Perktold, J. Cimrman, R.; Henriksen, I.; Quintero, E. A.; Harris, C. R.; Archibald, A. M.; Ribeiro, A. H.; Pedregosa, F.; van Mulbregt, P.; and SciPy 1.0 Contributors. SciPy (v1.13.1): Fundamental Algorithms for Scientific Comuputing in Python. **2020**. *Nature Methods*. DOI: 10.1038/s41592-019-0686-2.

(5) Richardson N.; Cook I.; Crane N.; Dunnington D.; François R.; Keane J.; Mecum B.; Moldovan-Grünfeld D.; Ooms J.; Wujciak-Jens J. Apache Arrow (v22.0.0). **2025**. arrow: Integration to 'Apache' 'Arrow'. Python package version 22.0.0, https://github.com/apache/arrow/.

(6) Whitaker, J. netCDF4 (version 1.7.2). **2025**. *Github*. Available at: https://github.com/Unidata/netcdf4-python.

(7) Jordahl, K.; Van den Bossche, J.; Fleischmann, M.; McBride, J.; Wasserman, J.; Gerard, J.; Garcia Badaracco, A.; Snow, A. D.; Tratner, J.; Perry, M.; Farmer, C.; Hjelle, G. A.; Cochran, M.; Gillies, S.; Culbertson, L.; Bartos, M.; Caria, G.; Eubank, N.; sangarshanan; Rey, S.; maxalbert; Bilogur, A.; Ward, B.; Ren, C.; Arribas-Bel, D.; Flavin; Wasser, L.; Wolf, L. J.; Journois, M.; abonte. geopandas/geopandas: v0.9.0 (v0.9.0). **2021**. *Zenodo*. DOI: 10.5281/zenodo.4569086

(8) Waskom, M. L. Seaborn: Statistical Data Visualization. *JOSS* **2021**, *6* (60), 3021. DOI: 10.21105/joss.03021.

(9) Hunter, J. D. Matplotlib: A 2D Graphics Environment. *Comput. Sci. Eng.* **2007**, *9* (3), 90-95. DOI: 10.1109/MCSE.2007.55.

(10) Crameri, F.; Shephard, G. E.; Heron, P. J. The Misuse of Colour in Science Communication. *Nat. Commun.* **2020**, *11*, 5444. DOI: 10.1038/s41467-020-19160-7

(11) California Air Resources Board. California Air District Boundaries, **2025**. https://gis.data.ca.gov/datasets/CaliforniaARB::california-air-district-boundaries/about (Accessed May 13, 2025).

(12) California Department of Conservation. California Important Farmland: 2016, **2016**. https://gis.conservation.ca.gov/portal/home/item.html?id=be4d1aff89824309b9300f4fdd3c64f2 (Accessed May 13, 2025).

(13) California Department of Pesticide Regulation. Pesticides Use Report, **2014**. https://calpip.cdpr.ca.gov/infodocs.cfm?page=navigate#navpur(Accessed May 13, 2025).

(14) California Department of Water Resources. Statewide Crop Mapping--California Natural Resources Agency Open Data, 2014, **2014**. https://data.cnra.ca.gov/dataset/statewide-crop-mapping (Accessed May 13, 2025).

(15) Kim, S.-Y.; Bechle, M.; Hankey, S.; Sheppard, L.; Szpiro, A. A.; Marshall, J. D. Concentrations of Criteria Pollutants in the Contiguous U.S., 1979 – 2015: Role of Prediction Model Parsimony in

Integrated Empirical Geographic Regression. *PLOS ONE* **2020**, *15* (2), e0228535. DOI: 10.1371/journal.pone.0228535.

(16) van Donkelaar, A.; Hammer, M. S.; Bindle, L.; Brauer, M.; Brook, J. R.; Garay, M. J.; Hsu, N. C.; Kalashnikova, O. V.; Kahn, R. A.; Lee, C.; Levy, R. C.; Lyapustin, A.; Sayer, A. M.; Martin, R. V. Monthly Global Estimates of Fine Particulate Matter and Their Uncertainty. *Environ. Sci. Technol.* **2021**, *55* (22), 15287–15300. DOI: 10.1021/acs.est.1c05309.
